# Structure of Human Cytomegalovirus UL141 Binding to TRAIL-R2 Reveals Novel, Non-canonical Death Receptor Interactions

**DOI:** 10.1371/journal.ppat.1003224

**Published:** 2013-03-21

**Authors:** Ivana Nemčovičová, Chris A. Benedict, Dirk M. Zajonc

**Affiliations:** 1 Division of Cell Biology, La Jolla Institute for Allergy and Immunology, La Jolla, California, United States of America; 2 Division of Immune Regulation, La Jolla Institute for Allergy and Immunology, La Jolla, California, United States of America; Washington University, United States of America

## Abstract

The TRAIL (TNF-related apoptosis inducing ligand) death receptors (DRs) of the tumor necrosis factor receptor superfamily (TNFRSF) can promote apoptosis and regulate antiviral immunity by maintaining immune homeostasis during infection. In turn, human cytomegalovirus (HCMV) expresses immunomodulatory proteins that down-regulate cell surface expression of TNFRSF members as well as poliovirus receptor-related proteins in an effort to inhibit host immune effector pathways that would lead to viral clearance. The UL141 glycoprotein of human cytomegalovirus inhibits host defenses by blocking cell surface expression of TRAIL DRs (by retention in ER) and poliovirus receptor CD155, a nectin-like Ig-fold molecule. Here we show that the immunomodulatory function of HCMV UL141 is associated with its ability to bind diverse proteins, while utilizing at least two distinct binding sites to selectively engage TRAIL DRs or CD155. Binding studies revealed high affinity interaction of UL141 with both TRAIL-R2 and CD155 and low affinity binding to TRAIL-R1. We determined the crystal structure of UL141 bound to TRAIL-R2 at 2.1 Å resolution, which revealed that UL141 forms a homodimer that engages two TRAIL-R2 monomers 90° apart to form a heterotetrameric complex. Our structural and biochemical data reveal that UL141 utilizes its Ig-domain to facilitate non-canonical death receptor interactions while UL141 partially mimics the binding site of TRAIL on TRAIL-R2, which we found to be distinct from that of CD155. Moreover, UL141 also binds to an additional surface patch on TRAIL-R2 that is distinct from the TRAIL binding site. Therefore, the breadth of UL141-mediated effects indicates that HCMV has evolved sophisticated strategies to evade the immune system by modulating multiple effector pathways.

## Author Summary

Natural killer (NK) cells are critical to the innate immune system along with their ability to detect and destroy cells infected by viruses. To avoid discovery by cytotoxic lymphocytes and to allow for longtime persistence in the host, human cytomegalovirus (HCMV) evolved a number of genes to evade or inhibit immune recognition. Expression of the HCMV UL141 glycoprotein could represent a unique mechanism to allow the virus to escape immune recognition as we show here that UL141 is capable of engaging diverse proteins containing disparate structural folds. UL141 utilizes at least two distinct binding sites to selectively engage TRAIL death receptors and the NK cell activating ligand CD155. Although it is common for immune modulatory proteins to have evolved to target a specific protein, or a family of host proteins, targeting diverse proteins that contain disparate folds is extremely rare. Therefore, structure-function analysis of pleiotropic UL141 and its interaction with cellular receptors will shed light upon mechanism how HCMV employs UL141 to inhibit immune recognition.

## Introduction

The immune system has evolved to protect against the many pathogens that are encountered throughout the lifetime of an individual. In turn, the selective pressure that is exerted by the immune system has shaped pathogen evolution. This co-evolutionary relationship between host and pathogen is particularly clear for viruses that establish persistent infections, such as human herpesviruses (HHV) [1], [2]. Human cytomegalovirus (HCMV, a β-herpesvirus, HHV-5), is a large double-stranded DNA virus that causes a lifelong, persistent/latent infection in ∼50–80% of the US population, varying with age, geography and socioeconomic status. While HCMV infection is largely asymptomatic in healthy persons, it can induce serious disease in those with naïve or compromised immunity, and the high incidence of congenital infection has spurred a strong initiative for vaccine development [3]. Primary clinical isolates carry at least 19 additional genes within the UL/b′ genomic region (UL133–151 locus) that have been lost in several commonly used HCMV strains that have been passaged extensively in tissue culture [4], [5], with several of them targeting signaling by the TNFR superfamily (e.g. UL144 and UL138) [6].

The interaction between TNF ligands and their respective TNFRs controls pleiotropic biological responses, including cell differentiation, proliferation and apoptosis [7]. Both TNF ligands and TNFRs are expressed on T cells and, as such, play important roles in T cell co-stimulation. In addition, TNF superfamily members are crucial in controlling herpesvirus infection by initiating the direct killing of infected cells and by enhancing immune responses [8], [9]. For instance, TRAIL death receptor (TRAIL-DR) regulation of apoptosis is critical for maintaining immune homeostasis during HHV infection. The herpesviruses, however, can block apoptosis, likely facilitating their ability to establish lifelong infection [10], [11]. Using specific genetic mutants of HCMV we have recently identified UL141 to restrict expression of TRAIL-DR (TRAIL-R1/DR4 and TRAIL-R2/DR5) [12]. We have shown that cells infected with an HCMVΔUL141 mutant are more susceptible to killing by TRAIL, and that UL141 is both necessary and sufficient to retain both TRAIL receptors in the ER, thus preventing their cell surface expression [12].

HCMV UL141 is also necessary and sufficient to inhibit cell surface expression of CD155 (PVR, poliovirus receptor; nectin-like molecule 5), a ligand for the NK cell activating receptor DNAM-1 (CD226). DNAM-1 also binds a second ligand, CD112 (nec-2, PRR-2, poliovirus receptor-related protein 2), and UL141 is required, but not sufficient, to target CD112 for proteasome-mediated degradation. As a consequence, both activating ligands for DNAM-1 are removed from the surface of HCMV infected cells, and NK cell killing of those cells is markedly inhibited [13], [14], [15], [16]. In addition, we have recently shown that UL141 inhibition of TRAIL DR also contributes to inhibit TRAIL-mediated NK cell killing [12].

Despite inducing a strong host immune response, HCMV persists for life in a latent form, which can be rapidly reactivated in the absence of host immunity, highlighting the dynamic relationship between the host and this virus. Characterizing the structural and molecular basis of the interactions that occur between specific HCMV proteins and the host molecules they target is crucial for our understanding of viral persistence, and will ultimately facilitate vaccine and antiviral drug development. Here, we report the structural and biochemical characterization of the novel, non-canonical interaction between UL141 and TRAIL-R2, an interaction that has evolved to inhibit cell death mediated by TRAIL signaling and mute host defenses. Remarkably, UL141 displays no structural homology to TNF superfamily ligands, and instead utilizes its Ig-domain to bind with high affinity to the TRAIL death receptor. To elucidate how UL141 is capable of engaging diverse proteins containing disparate structural folds, we have characterized the binding interactions of UL141 with both TRAIL-R1 and TRAIL-R2, as well as CD155, by surface plasmon resonance. Finally, the crystal structure of UL141 bound to TRAIL-R2 was solved at a resolution of 2.1 Å, allowing the differential comparison of how TRAIL-R2 binds to both TRAIL and UL141.

## Results

### Binding of UL141 to TRAIL death receptors and CD155

Recently, we have shown the UL141 protein of HCMV is both necessary and sufficient to inhibit cell surface expression of the TRAIL death receptors and that UL141 can bind directly to the ectodomain of TRAIL-R1 and TRAIL-R2 [12]. This discovery revealed an unexpected pleiotropic role of UL141 in regulating host immunity, as previously this HCMV protein was known to only target the nectin-related molecules CD155 and CD112. TRAIL, is highly expressed by activated immune effector cells and can mediated apoptosis [17], [18], [19], and UL141 restriction of TRAIL DR expression [12] likely contributes to its role as a potent NK cell inhibitor. As UL141 is sufficient to restrict expression of both CD155 and the TRAIL DRs, we sat out to determine the relative binding affinities of UL141 for these host cell proteins. All binding partners were produced as Fc fusion proteins, with an engineered protease cleavage site allowing for the release of the individual ectodomains. The monovalent binding interactions were then analyzed by Surface Plasmon Resonance (SPR), while the Fc fusion proteins were immobilized on the sensor chip. Recombinant UL141 bound directly to TRAIL-R2-Fc and CD155-Fc (with high affinity (K_D_ of 6 nM [12] and 2 nM, respectively) (Table 1). When UL141-Fc was immobilized on the sensor chip and the binding of TRAIL-R2 was assessed, the dissociation rate was roughly 80-fold faster, while association was also 20-fold faster, leading to a 3.5-fold lower equilibrium binding affinity (K_D_ = 21.4 vs. 6 nM, Table 1). The change in binding kinetics suggested that UL141 is not a monomer in solution. This increased avidity is also in agreement with size exclusion chromatography results showing UL141 is a non-covalently associated dimer in solution, while recombinant TRAIL-R2 is a monomer (Figure S1a, b). Interestingly, the binding kinetics of UL141 to either TRAIL-R2 or CD155 differed significantly (Table 1). UL141 bound to CD155 with a 14-fold faster association rate (k_a_, k_on_), while dissociation was 5-times faster (k_d_, k_off_), resulting in a nearly 3-fold higher equilibrium binding affinity (K_D_). The observed kinetic differences indicated that UL141 uses either distinct binding sites to bind TRAIL-R2 and CD155, or suggested different binding mechanisms (e.g. induced fit versus lock-and-key). To test these hypotheses, the high-affinity UL141–TRAIL-R2 complex was pre-formed, and binding to CD155-Fc was assessed by SPR (Table 1). The binding kinetics of the UL141-TRAIL-R2 complex to CD155-Fc showed no difference from that of soluble UL141, strongly suggesting that the UL141 binding sites for TRAIL-R2 and CD155 are largely distinct. We further confirmed this by a sequential binding experiment. CD155-Fc was immobilized on the sensor chip and UL141 was then injected and bound to CD155. Subsequent injection of TRAIL-R2 lead to additional binding to the CD155-Fc/UL141 complex, demonstrating that UL141 can bind to both receptors simultaneously (Figure S2). Although, TRAIL-R1 is highly homologous in primary sequence to TRAIL-R2, the binding affinity of UL141 for this DR was found to be ∼400-fold reduced (K_D_ = 2.3 µM) when compared to TRAIL-R2 (K_D_ = 6 nM) [12]. The binding kinetics revealed a 2-fold slower association rate (k_a_, k_on_), while dissociation was almost 200-times faster (k_d_, k_off_) when compared to UL141 binding to TRAIL-R2-Fc (Table 1). The rapid dissociation from TRAIL-R1 suggests that UL141 has a less optimized binding surface for TRAIL-R1, and instead has evolved to preferentially target TRAIL-R2.

**Table 1:** Binding kinetics measured by SPR.

	Immobilized (ligand)	In solution (analyte)	K_Deq_ [M]	χ	R_max_	K_D_ [M]	k_on_ [M^−1^s^−1^]	k_off_ [s^−1^]	K_Dave_ b) [nM]
A	UL141–Fc	TRAIL-R2	19.8×10^−9^	0.71	39.9	21.4×10^−9^	2.64×10^5^	5.64×10^−3^	20 nM
B	TRAIL-R2–Fc	UL141	n.d.a)	1.33	41.2	5.96×10^−9^	1.21×10^4^	7.21×10^−5^	6 nMc)
C	TRAIL-R1–Fc	UL141	2.27×10^−6^	0.88	40.2	2.33×10^−6^	6.02×10^3^	1.40×10^−2^	2.3 µMc)
D	CD155–Fc	UL141	n.d.a)	2.11	31.3	1.97×10^−9^	1.76×10^5^	3.46×10^−4^	2 nM
E	CD155–Fc	UL141–TRAIL-R2	n.d.a)	1.98	87.1	2.19×10^−9^	1.52×10^5^	3.33×10^−4^	2 nM

a) Samples with low analyte concentrations did not reach chemical equilibrium (plateau phase) during injection, which is required to perform a reliable steady-state analysis (K_Deq_).

b) Average equilibrium binding affinity (K_Dave_) was derived from both K_Deq_ and K_D_.

c) values from ref. [12].

### UL141–TRAIL-R2 complex structure determination

**Figure 1:**
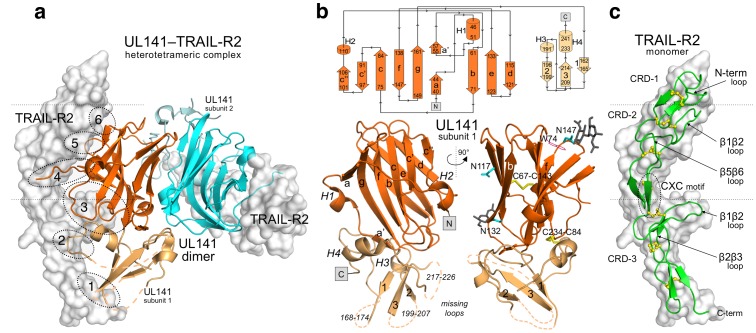
Crystal structure of the UL141–TRAIL-R2 complex. (**a**) Hetero-tetrameric structure of the UL141 dimer (orange and cyan cartoon) in complex with two TRAIL-R2 monomers (grey surface). Six distinct binding patches between UL141 and TRAIL-R2 are indicated with dotted circles. (**b**) 2-D topology diagram (generated by PDBsum [56]) of the UL141 subunit with arrows from N-terminus to C-terminus and two cartoon representations of one UL141 subunit 90° rotated. The N-terminal domain of UL141 exhibits a V-type immunoglobulin superfamily fold containing ten β-strands (a–g) and two short α-helices H1 and H2 (orange). The C-terminal domain contains three β-strands (1–3) and two short α-helices H3 and H4 (light orange). Disulfide bonds (C84–C234 and C67–C143) are indicated as yellow sticks. Potential N-linked asparagines are drawn as cyan sticks and glycans are shown in grey for one UL141 monomer (chain A) at position N132 and N147, while N117 is occupied in the other monomer (chain B). Disordered loops 168–174, 199–207 and 217–226 are indicated as dotted lines. (**c**) The cartoon representation of TRAIL-R2 structure colored green with molecular surface in grey. Dotted lines delineate CRDs (Cysteine Reach Domains); CRD-1 (residues 78–94), CRD-2 (residues 95–137) and CRD-3 (residues 138–178). Disulfide bonds are depicted in yellow as ball-and-sticks. The β1β2 loop of CRD-3 (residues 143–157) and that of CRD-2 (residues 104–115) that make the key contacts with the ligands as well as other important loops (β5β6 of CRD-2, β2β3 of CRD-3, N-term loop) and CXC motif are highlighted by circle or arrows.

The complex of HCMV UL141 (residues 30–279) bound to human TRAIL-R2 (residues 58–184, both numberings start from the initial methionine) was crystallized and the structure determined by single anomalous dispersion (SAD), using experimental phases derived from selenomethionine labeled protein expressed in Sf9 insect cells (see METHODS) (Table 2). The crystal structure was refined to a resolution of 2.1 Å with an R factor of 22.3% and an R_free_ of 27.4%. With the exception of several mobile loops of UL141, the entire N-terminal Ig-like β-sandwich domain and the cysteine rich domain (CRD) of TRAIL-R2 (starting at residue 75–182) are well ordered. One UL141 dimer binds two TRAIL-R2 monomers through non-crystallographic two-fold symmetry resulting in a heterotetrameric complex (Figure 1a).

**Table 2:** Data collection and refinement statistics.

	UL141–TRAIL-R2	UL141–TRAIL-R2	UL141–TRAIL-R2
	native	C6 crystal derivative	Multi-crystalb) derivative (including C6 data)
**Data collection statistics**			
Space group	*P2_1_2_1_2_1_*	*P2_1_2_1_2_1_*	*P2_1_2_1_2_1_*
Cell dimension			
*a*, *b*, *c* (Å)	67.7, 97.7, 141.3	67.9, 97.0, 141.4	67.9, 97.0, 141.6
α, β, γ (°)	90.0, 90.0, 90.0	90.0, 90.0, 90.0	90.0, 90.0, 90.0
Resolution range (Å)	65.1-2.5	51.8-2.3	19.8-2.1
a) [outer shell]	[2.8-2.5]	[2.3-2.3]	[2.2-2.1]
Wavelength (Å)	0.9698	0.9795	0.9795
No. reflections	33185	40153	100305
R_merge_ (%)	8.1 [69.2]	9.2 [65.3]	10.4 [66.6]
Multiplicity	5.3 [5.4]	7.4 [7.5]	7.5 [10.3]
Average I/σ(I)	13.2 [3.1]	8.9 [2.5]	8.4 [2.1]
Completeness (%)	100.0 [100.0]	99.9 [100.0]	99.4 [97.5]
**Refinement statistics**			
No. Atoms	-	-	4999
Protein	-	-	4685
Carbohydrate	-	-	70
Waters	-	-	239
Other solvent	-	-	5
R_work_/R_free_ (1.8%)	-	-	0.223/0.274
Ramachandran plot (%)	-	-	
Favored	-	-	95.6
Allowed	-	-	99.6
R.m.s. deviations	-	-	
Bonds (Å)	-	-	0.013
Angles (°)	-	-	1.450
*B*-factors (Å^2^)	-	-	
Protein	-	-	51.0
Carbohydrate	-	-	52.8
Waters	-	-	54.5
Other solvent	-	-	43.9

a) Values in parenthesis refer to highest resolution shell,

b) Multi-crystal data contain all five merged datasets.

### Structure of UL141

UL141 has no sequence similarity to any other known cellular protein. For SPR studies, the UL141 ectodomain was expressed as a thrombin-cleavable Fc fusion protein in *Spodoptera frugiperda* (Sf9) insect cells using the baculovirus mediated expression system. However, recombinant UL141 purified by this method gradually lost its ability to bind to TRAIL-R2 within 3 days, suggesting it was unstable in solution. As an attempt to attain stable and homogeneously glycosylated protein for structural studies, UL141 was co-expressed with TRAIL-R2 in Sf9 cells. The resulting complex was co-purified, and was found to be stable in solution for several weeks and amenable to crystallization. In agreement with our biochemical analysis using size exclusion chromatography, UL141 forms a non-covalent homodimer (Figure S1a and Figure 1a). Structural analysis revealed that UL141 interacts in a head-to-tail fashion to form a well-packed dimer that is stabilized primarily through polar interactions, such as hydrogen bonds and salt bridges, and several hydrophobic contacts, while burying a total surface area of 1423 Å^2^. The UL141 ectodomain exhibits an N-terminal immunoglobulin (Ig)-like domain, followed by an additional C-terminal β-sheet domain (Figure 1a, 1b). The presence of ten β-strands, arranged in two antiparallel β-sheets (formed by β-strands a, a′, g, f, c, c′, c″ and β-strands d, e, b, respectively) and a tryptophan residue (W74) packed over a central disulfide bond (C67–C143) linking β-strands b and f clearly classifies it as a variable (V-type) Ig-like domain (residues 32–161). The additional C-terminal β-sheet domain (amino acids 162–246) is formed by a three-stranded antiparallel β-sheet (β-strands 1, 2 and 3) and two short α-helices at the C-terminus (H3 residues 191–195, H4 residues 233–241). The N-terminal Ig-like domain also features two additional short α-helices (H1 and H2). Helix H1 (residues 46–51) separates the β-strand a from a′ and the ‘one-turn’ helix H2 (residues 107–110) is between c″ and d β-strands. The second disulfide bond of UL141 (C84–C234) connects α-helix H4 of the C-terminal domain with the bottom of the N-terminal Ig-like domain.

### Structure of the TRAIL-R2 human death receptor

**Figure 2:**
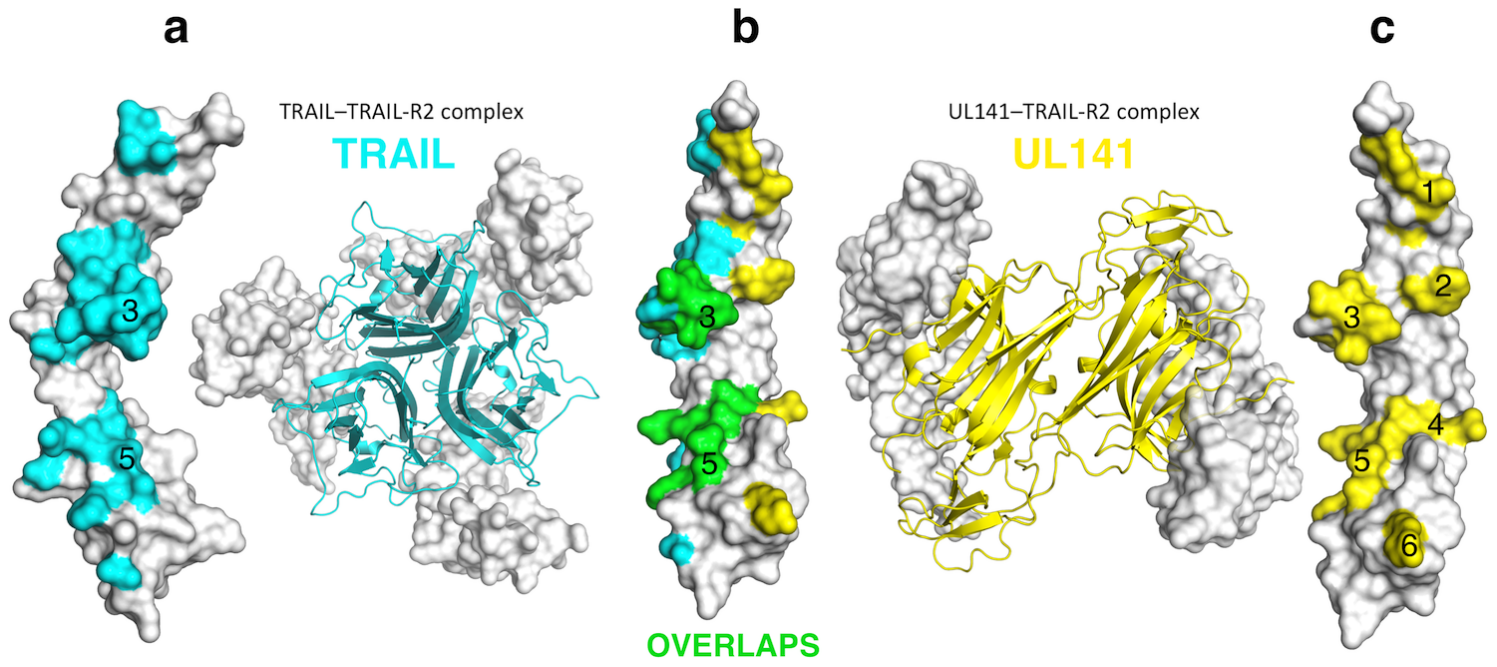
Comparison of the UL141 and TRAIL binding footprints on TRAIL-R2. TRAIL-R2 is shown as a grey molecular surface in three different orientations: left (**a**), front (**b**), and right (**c**). The binding interface between the UL141 and TRAIL-R2 (**c**) is divided into six binding patches, with patches 3 and 5 being similar to that of the TRAIL–TRAIL-R2 complex (**a**). TRAIL contact residues on TRAIL-R2 in cyan (**a–b**), UL141 contact residues on TRAIL-R2 in yellow (**b–c**), while the overlapping residues are green (**b**).

The structure of TRAIL-R2 bound to its homotrimeric cellular ligand TRAIL has been reported previously ([20] PDB: 1DU3; [21] PDB: 1D4V; [22] PDB: 1D0G). Each monomer of the trimeric TRAIL, binds to one TRAIL-R2 molecule, thereby leading to the trimerization and clustering of TRAIL-R2 on the cell surface, the hallmark oligomerization state thought to initiate signaling by TNFRs (Figure 2a).

TRAIL-R2 is a monomer in solution (Figure S1b) with structurally conserved features of other members of the TNFR superfamily. It adopts an elongated structure composed of three extracellular pseudorepeats, or CRD's (Cysteine-Rich Domain), characterized by a cysteine knot topology [23], [24]. CRD-1–3 span a length of 70 Å, and CRD-2 and CRD-3 form the major ligand-binding region in the UL141–TRAIL-R2 complex (Figure 1a, 1c). CRD-1 of TRAIL-R2 is incomplete as it contains only a single disulfide bond while CRD-2 and -3 of TRAIL-R2 correspond to the central two repeats of other TNFRSFs. Together, CRD-2 and CRD-3 form the binding interface for LTα–TNFR-1, TNFα–TNFR-2, RANKL-RANK as well as for TRAIL–TRAIL-R2. These two ligand-binding repeats are joined in all TNFRSF molecules by a CXC motif (CQC in all the TRAIL receptors, CGC in TNFR-1, CTC in TNFR-2 and CAC in RANK) (Figure 1c and Figure S3) that acts as a flexible articulation point.

### UL141–TRAIL-R2 complex architecture

**Figure 3:**
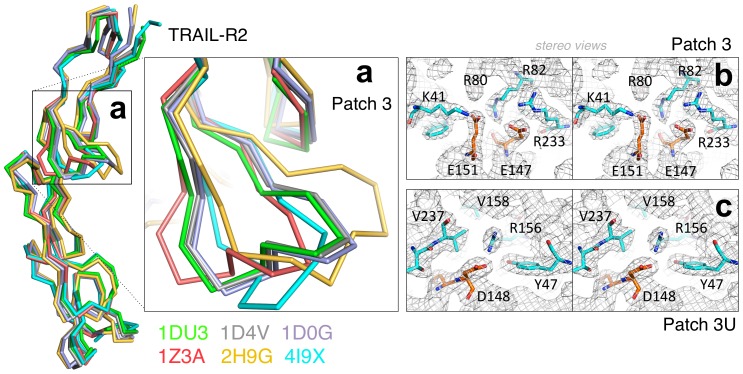
Comparison of TRAIL-R2 structures. The TRAIL-R2 structure derived from the complex with UL141 (4I9X, in cyan) is superimposed (residues 78–178) with available TRAIL-R2 crystal structures from PDB database. Three TRAIL-R2–TRAIL structures: 1D4V (grey), 1DU3 (green) and 1D0G (light purple) and two TRAIL-R2–Fab structures: YSd1 Fab (1Z3A, red) and BdF1 Fab (2H9G, yellow). (**a**) Structures superimpose very well with the exception of the β1β2 loop of CRD-3 (Patch 3). Representative 2F_O_-F_C_ electron density map contoured at 1σ, showing the key residues of receptor β1β2 loop (cyan) interacting with UL141 residues (orange) in patch 3 (**b**) and patch 3 U (**c**) in stereo views. The well-defined electron density indicates, that the β1β2 loop of CRD-3 is well ordered upon UL141 binding.

In contrast to TRAIL binding to TRAIL-R2, which leads to head-to-head (180°) trimerization of the receptor, UL141 binds both TRAIL-R2 monomers diagonally across the entire UL141 ectodomain, resulting in an approximate rotation of both TRAIL-R2 monomers of 90° to each other (Figures 1, 2 and Figure S4). The high affinity interaction between UL141 and TRAIL-R2 correlates well with the large solvent-accessible surface area of the heterotetrameric complex in which 2694 Å^2^ are buried on the UL141 dimer (1347 Å^2^ per monomer) and 1351 Å^2^ are buried on each TRAIL-R2 monomer. The large binding surface on each TRAIL-R2 subunit is further concentrated in three binding regions (upper, lower, and central region) that can further be divided into six distinct binding patches based on our mutational data (Figures 1 and 2). The upper binding region contains patch 6, the central region patches 4 and 5, while the lower binding region combines patches 1, 2, 3 (Figure 2). Structural comparison with the TRAIL-TRAIL-R2 complex (PDB: 1D4V, 1DU3 and 1D0G) reveals that patches 3–5 on TRAIL-R2 partially overlap with the binding site for the endogenous ligand TRAIL, while patches 1, 2, 6, and part of patch 3 are unique to the binding of UL141. Two structures of TRAIL-R2 in complex with bound Fab's had also been determined earlier ([25] PDB: 2H9G; [26] PDB: 1ZA3), allowing the structural comparison of TRAIL-R2 from multiple receptor-ligand complexes. Superimposition of all five TRAIL-R2 structures revealed structural changes within TRAIL-R2 that likely result from binding to distinct ligands (Figure 3). This structural change is located in the central binding region of TRAIL-R2 (CRD-3 β1β2 loop, residues 143–157, part of patch 3). Superposition of CRD-3 β1β2 loop (15 Cα atoms) resulted in a root-mean-square deviation (rmsd) of 0.19 Å and 0.38 Å when PDB ID 1D4V was aligned with 1D0G and 1DU3, respectively. The β1β2 loop is well conserved among all previously published TRAIL–TRAIL-R2 complexes (grey, green, light purple), but adopts different orientations upon binding of distinct antibodies (rmsd of 1.54 Å for 1ZA3 in red and 0.29 Å for 2H9G in yellow) or UL141 (cyan, rmsd of 2.02 Å).

### TRAIL-R2 binding site analysis

**Figure 4:**
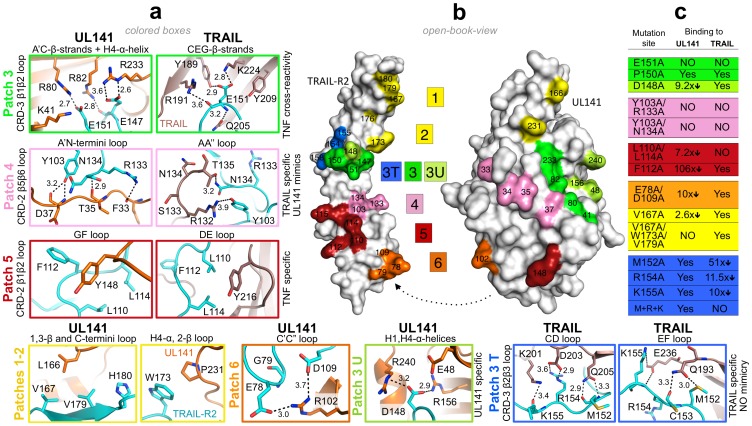
Comparison of receptor-ligand interaction between UL141 and TRAIL with TRAIL-R2 and mutational binding data. (**a**) Detailed interaction is shown for the six binding patches of the UL141–TRAIL-R2 complex; 1–2 (yellow), 3 (green), 3 U (light-green), 4 (pink), 5 (red), 6 (orange), as well as for the patches 3, 3T (blue), 4 and 5 of the TRAIL–TRAIL-R2 complex (same coloring scheme). Interaction residues are labeled and drawn as orange (UL141), salmon (TRAIL) and cyan (TRAIL-R2) sticks with atoms colored as follows: nitrogen (blue), oxygen (red) and sulfur (yellow). The molecular contacts (hydrogen bonds and salt bridges with distance <4.0 Å) are shown as dashed black lines. The name of interacting loop, helix or strand is listed around each box as well as the specificity of particular contact patch. (**b**) Open book view of UL141–TRAIL-R2 complex with their molecular surfaces outlined in grey. All binding patches (fingerprints on both molecules) follow the same color-code as above including residues selected for alanine scanning mutagenesis. (**c**) Relative effect on alanine mutagenesis of TRAIL-R2 on UL141 (middle column) and TRAIL (right column) binding, as analyzed by SPR (Figure 5 and Table S1). Mutated residues are listed (left column). Mutation that do not affect receptor binding are labeled ‘YES’ while ‘NO’ indicates binding is abrogated. X-fold reduction in binding (compared to wild-type) is quantitated by numbered arrows.

Based on the contact residues identified in the UL141-TRAIL-R2 complex, alanine-scanning mutagenesis of TRAIL-R2 was then performed followed by SPR analysis to assess the relative binding requirements for TRAIL and UL141 (Figure 2 and 4). Notably, all six binding patches contain residues that contribute to the binding of UL141 and/or TRAIL. In the following results sections, we report how this mutagenesis analysis has revealed that UL141 has evolved to bind uniquely to this TRAIL DR:

### UL141 interacts with TRAIL-R2 in a unique fashion

**Figure 5:**
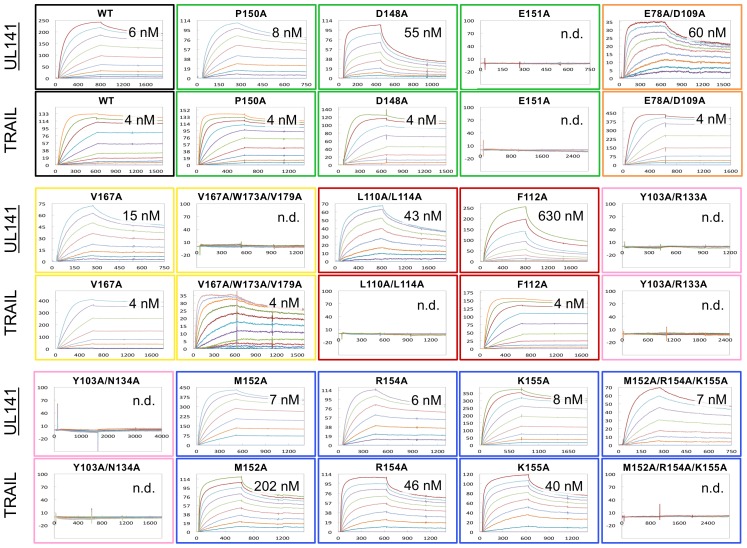
Binding of TRAIL-R2 mutants to TRAIL and UL141. Surface plasmon resonance study to assess the binding contribution of individual TRAIL-R2 residues to both viral UL141 and endogenous TRAIL. A Sensorgram for each kinetics experiment is shown in colored boxes (colored by binding patch as in Figure 4). The specific alanine mutation on TRAIL-R2 as well as the calculated binding constant K_D_ (nM) are indicated for each panel. Mutations that fully abrogate binding are indicated as n.d. (binding not detected).

In the UL141–TRAIL-R2 complex, unique contacts are formed involving E78 and D109 of TRAIL-R2 that form two salt-bridges with R102 of UL141 (Figure 4, Patch 6). TRAIL-R2 mutation D109A together with E78A reduced UL141 binding affinity 10-fold, while having no effect on TRAIL-binding (Figure 5). In addition, D148 of TRAIL-R2 receptor (Figure 3c) forms two salt-bridges with R240 and R156 (helix H4 and g β-strand, respectively) of UL141 (Figure 4, Patch 3U). The mutation D148A on TRAIL-R2 lead to a 10-fold reduced binding affinity for UL141 (K_D_ = 55 nM, Figures 4c and 5, Table S1). In addition, the C-terminal loop of TRAIL-R2 (V167, V179 and W173, Figure 4, Patch 1–2) is slightly pulled toward UL141 compared to those of other TRAIL-R2 structures (main chain – main chain distance difference is 1.8–2.1 Å), as it forms several contacts with UL141 (L166, Y248 and P231, Figure 4, Patch 1–2). The interactions within this binding region 1 (patches 1–2) are hydrophobic, in contrast to the centrally located patches 2–4, which are dominated by electrostatic interactions. Among the hydrophobic interface residues, the TRAIL-R2 V167A mutant exhibits a 2.5-fold reduced binding affinity to UL141 (K_D_ = 15 nM), while the triple mutation (V167A-W173A-V179A) abolishes binding to UL141 completely. Strikingly, all these mutations in binding region 1 are unique to UL141, having no effect on TRAIL binding (Figures 4c and 5, Table S1).

### UL141 mimics several TRAIL-specific interactions

The central binding interface of the UL141-TRAIL-R2 complex is structurally similar to other TNF-TNFR complexes (Figure 4, Patch 4), and involves residues 33–37 of UL141 that correspond to TRAIL residues 131–135 (A′N-termini loop connecting strand a′ with the N-terminus of UL141; called AA″ loop in TNF ligands). This binding loop forms several specific polar interactions with CRD-2 β1β2 and β5β6 loop of TRAIL-R2, displaying well-ordered electron density (not shown). Y103 forms a hydrogen bond with D37 in UL141 while the same Y103 forms a polar interaction with the guanidino group of R132 in TRAIL. N134 of TRAIL-R2 interacts with T35 and T135 of UL141 and TRAIL, respectively. R133 forms a hydrogen bond with the main chain oxygen of F33 of UL141 while it forms no contact with TRAIL. These three interacting residues (Y103, R133 and N134) are also conserved in their nature in the other three TRAIL receptors, and this is shown by sequence alignments of several TNFRSF members (Figure S3b, framed pink). However, the AA″ loop in RANKL folds toward the top third of the molecule and is positioned above the β2β3 loop of the RANK receptor, whereas the same loop in LTα is very short and does not make any interaction with TNFRSF1A [23], [27]. Our mutagenesis data confirmed that these interactions (in patch 4, pink) are crucial for TRAIL binding and mimicked by UL141, as alanine mutations in this region of TRAIL-R2 completely abolished binding to both UL141 and TRAIL (Figure 4c, pink). Moreover, deleting the AA″ loop in TRAIL completely abolishes its biological activity [21]. The combined structural and mutational data suggest that contact patch 4 is specific and crucial for TRAIL ligand binding and that viral UL141 mimics this structural motif to specifically engage this TRAIL DR.

### UL141 mimics a hydrophobic binding motif utilized by TNF-family ligands

In addition to UL141 mimicking the electrostatic interaction of TRAIL with TRAIL-R2 through the use of binding patch 4, UL141 also mimics a ‘TNF-specific’ hydrophobic binding motif located within binding patch 5 in the central region of CRD-2 (Figure 4, Patch 5). This patch on TRAIL-R2 is formed by the hydrophobic residues of the β1β2 loop of CRD-2 (L110, L114 and F112) that cluster around Y148 of the GF loop of UL141 (connecting β-strands g and f). Similar interactions are formed by TRAIL, which utilizes Y216 to interact with L110 and L114 but not F112 of TRAIL-R2. These contacts are also conserved within other TNF-TNFR complexes and include Y108 in LTα, and I248 in RANKL (called DE loop in TNF ligands) (Figure S3, red box). The aromatic interaction formed between Y148 of UL141 and F112 of TRAIL-R2 is critical for maintaining a stable complex, as the F112A mutation of TRAIL-R2 results in a 100-fold decrease in binding affinity (K_D_ = 630 nM). In contrast, the F112A mutation does not affect TRAIL binding. However, double mutation of L110A and L114A abolished binding to TRAIL completely, while only a 7-fold decrease in binding affinity was observed for UL141 (K_D_ = 43 nM). Therefore, the aromatic interaction involving F112 of TRAIL-R2 (which does not form a contact with TRAIL) dominates this binding interface with UL141, while TRAIL binding depends strongly on the hydrophobic interaction with both L110 and L114 of TRAIL-R2. In addition, both leucines are conserved or substituted with similar amino acids in other TNF-TNFR complexes (L110/L114 in TRAIL-TRAIL-R2, L67/L71 in LTα-TNFR1, Figure S3b in red box). Moreover, Y216 in TRAIL has been identified by alanine scanning mutagenesis as a critical residue for bioactivity and receptor binding [22] and sequence comparison indicates its conservation in many of the TNF superfamily ligands including TRAIL, RANKL, TNFα, LTα and FasL (Figure S3a). Others have also showed the importance of this tyrosine, where mutation in TRAIL, RANKL, TNFα, LTα and FasL abolished receptor binding [21], [28], [29], [30], [31]. In summary, patch 5 involves strong hydrophobic features important for the stability of complexes throughout the TNF/TNFR superfamily, and we postulate that UL141 has evolved to mimic this interaction in order to modulate this TRAIL DR.

### Control of cross-reactivity between TNF superfamily members

Patch 3 of TRAIL-R2 forms the most intensive interaction in the central to upper binding region with UL141. The contacts are maintained by CRD-3 β1β2 loop of TRAIL-R2, which interacts with a positively charged cluster of UL141 residues centered around K41, R80 and R82 of strands a and c, as well as R233 of helix H4 (Figure 3b). Consequently, a positively charged pocket is formed by UL141 that engages the negatively charged glutamic acid residues of TRAIL-R2 (E151 and E147) through several salt bridges (Figure 4, Patch 3, green). Sequence alignment reveals conservation of this region in TNFRSF10A–D, which covers all four TRAIL receptors, whereas the contacting residues of the cognate TNF ligands are spread across the entire sequence (Figure S3, green boxes).

In contrast to the UL141 interacting residues of patch 5, no residues within patch 3 are conserved in the other three TNF-TNFR complexes (Figure S3), highlighting the complexity of the ligand-receptor binding in the superfamily. Moreover, we performed mutagenesis in the participating β1β2 loop of the receptor and found that mutation E151A had the most dramatic effect on both UL141 and TRAIL binding (no binding in SPR with up to 1 µM ligand). Therefore, the electrostatic network contained within patch 3 contributes to the binding specificity and stability and likely controls cross-reactivity among the different TNF superfamily members as well as ligand recognition.

### TRAIL specific contacts within TRAIL-R2 not mimicked by UL141

Patch 3T is adjacent to patch 3, but exclusively contacts TRAIL (Figure 4, Patch 3T). It is maintained mostly by hydrogen-bonds and salt-bridges within a range of 2.8–4.5 Å. Two separate TRAIL monomers from the homotrimer (CD loop in first subunit and EF loop of the second subunit) contact the CRD-3 β2β3 loop of TRAIL-R2. This patch was first identified in TRAIL–TRAIL-R2 complex as a major binding area [20], [22] and it was reported that the CD and EF loops are disordered in the unbound TRAIL structure, while becoming ordered upon binding to TRAIL-R2. Alanine scanning of the TRAIL-R2 residues contained within patch 3T confirmed no effect on UL141 binding, while drastically reducing or abolishing TRAIL binding (Figure 4c). The TRAIL Q205A mutant had previously been reported to have a 700-fold reduced binding affinity for TRAIL-R2 [22] and we have further extended this mutational analysis by looking at the TRAIL-R2 interface. Alanine scanning of residues M152, R154 and K155 abolished binding to TRAIL ectodomain completely, while having no effect on UL141 binding. While M152 bridges both TRAIL subunits, the adjacent K155 and R154 of TRAIL-R2 form most contacts with D203 and K201 of the opposing TRAIL subunit. The TRAIL-R2 M152A mutant reduced binding affinity to TRAIL by ∼50-fold (K_D_ = 202 nM), while R154A (K_D_ = 46 nM) and K155A (K_D_ = 40 nM) mutants resulted in ∼10-fold weaker TRAIL binding. None of these TRAIL-R2 mutations affected UL141 binding. The receptors residues interacting in this patch (CRD-3 β1β2 loop) with the ligand may have an important role in controlling the specificity and cross-reactivity among the different TNF superfamily members, and therefore in ligand recognition, as these residues were not conserved in TNF ligand sequences (Figure S3).

### Accessible surface for receptor binding on UL141

**Figure 6:**
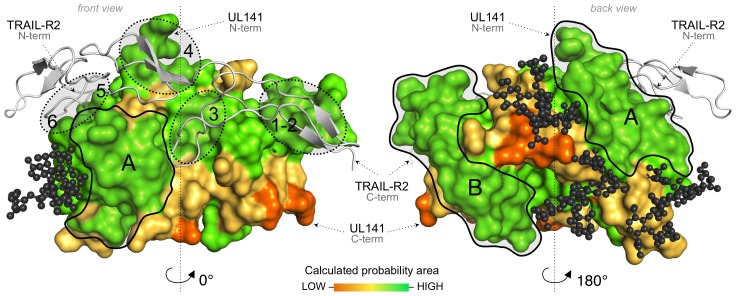
UL141 surface accessibility for receptor binding. Structure of one UL141 subunit (colored surface) in complex with one TRAIL-R2 (grey cartoon) shown in two views (0° and 180° turn). All three potential N-linked glycosylation sites (Asn117, Asn132 and Asn147) where modeled with a five-sugar Man_2_GluNac_2_Fuc glycans, shown in dark grey ball-and-stick). Area **A** and **B** indicate available and accessible protein binding sites on UL141, while other available areas are expected to be mostly covered with glycans in the fully glycosylated protein. Location of potential protein-protein binding sites for unbound UL141 were calculated using ProMate (http://bioinfo.weizmann.ac.il/promate). For simplicity, only one UL141 subunit is shown here in molecular surface colored from orange reflecting the lowest probability assigned, to green, assigned to the highest probability. The highest probability areas that reflect possible binding sites in UL141; excluding those binding sites 1–6 of TRAIL-R2 (shown in dotted line here); and are not shield by glycans, are areas **A** and **B**.

The UL141–TRAIL-R2 complex was not deglycosylated prior to crystallization, and all three putative N-glycosylation sites of UL141 display well-ordered electron density for N-linked carbohydrates (Asn117 and Asn147 of first subunit and Asn132 and Asn147 of the second subunit contained ordered carbohydrates). Modeling experiments predict that native, high-mannose glycosylation would not shield much of the UL141 surface from solvent, leaving ample space for binding to other ligands, such as CD155, assuming that complex glycans would project further outward into solvent (Figure 6). Importantly, our experimental data indicate that UL141 can simultaneously bind to both TRAIL-R2 and CD155 (see Table 1, Figure S2 and Figure S5), indicating the binding of multiple cellular proteins by a single UL141 dimer may have physiological relevance. Only the top of the (a, g, f, c, c′, c″)-β-sheet, as well as the front side of the C-terminal domain are expected to be largely covered with sugar in the fully glycosylated protein. For example, the solvent-exposed face of the (a, g, f, c, c′, c″)-β-sheet, the back face of C-terminal (1, 2, 3)-β-sheet domain and all four α-helices H1–4 are devoid of glycans and available for other potential interactions. In addition, we have calculated predictions for the location of potential protein-protein binding sites for unbound UL141 using the ProMate server (http://bioinfo.weizmann.ac.il/promate) (Figure 6). Interestingly, the highest probability binding area on UL141 is located on the back of the C-terminal domain (patch B), as well as the N-terminal Ig-like domain (patch A), which is formed by the surface exposed (c″, c′, c)-β-strands and the two α-helices (H2 and H4) (Figure 1 and 6). The highest probability was also calculated for the actual TRAIL-R2 binding sites on UL141, thereby validating the computational prediction by Promate. This analysis reveals that UL141 has two additional and separate binding sites that are suitable for protein binding. Supported by our competition binding data (Figure S2) indicating that TRAIL-R2 does not compete with CD155 for UL141 binding, we hypothesize that UL141 uses one of those two distinct surface-exposed binding sites within its two domains to bind to other proteins, such as the Ig superfamily member CD155. Recently, the crystal structure of another V-set Ig molecule TIGIT, bound to CD155 [32] has been determined. As UL141 recapitulates some of the structural features of TIGIT that are necessary for CD155 binding, superimposition of UL141 on TIGIT indicates that the potential binding site for CD155 on UL141 is indeed distinct from that of TRAIL-R2 and falls into the highest probability area A calculated by ProMate (Figure 6).

## Discussion

Human cytomegalovirus encodes several genes tightly linked to UL141 in the UL/b′ region that modulate host immune responses mediated by TNF-family proteins. These include UL138, which has recently been shown to promote the expression of TNFR-1, and UL144, a partial-mimic of HVEM (herpesvirus entry mediator) that exclusively binds the inhibitory receptor BTLA (B- and T-lymphocyte attenuator) [33]. Although it is common for herpesvirus immune modulatory proteins to have evolved to target a specific protein, or a family of host proteins, targeting diverse proteins that contain unique folds is rare. We now add the modulation of the TRAIL DRs to the arsenal of UL141 immune modulatory activity, in addition to its previously known role in restricting CD155 and CD112 expression. TRAIL DRs and CD155 belong to two structurally distinct families, the classical TNF receptor superfamily and the nectin-like Ig superfamily, respectively. While the only currently known natural ligand, TRAIL, belongs to the TNF superfamily, our structural analysis shows that UL141 assumes an Ig-like fold and shows no structural homology to TRAIL. However, UL141 does mimic key TRAIL binding motifs of TRAIL to TRAIL-R2, even though the Ig-fold of UL141 is entirely different from the homo-trimeric fold of TRAIL and other TNF ligands. Since UL141 and the Ig-like poliovirus receptors share no primary sequence homology, we favor the view that UL141 evolved independently, mimicking the central binding motif of TRAIL in addition to an as-yet unidentified binding motif to engage CD155.

Our structural and biochemical data further reveal that the TRAIL-R2 binding site on UL141 only partially overlaps with that of the endogenous ligand TRAIL, and appears to be entirely distinct from that which interacts with CD155 (Figure 2 and Table 1). Due to the strong sequence similarity of the TRAIL DRs, one might propose that TRAIL-R1 is likely to bind UL141 similarly to TRAIL-R2. However, the binding affinity of TRAIL-R1 is ∼400-fold reduced compared to that of TRAIL-R2, similar to the large differences in binding affinity that have been observed for TRAIL binding to its two death receptors [34]. In lack of a TRAIL-R1 crystal structure, we analyzed the sequence-similarities between TRAIL-R1 and -R2 and based on the sequence conservation between TNFR-fold proteins, and the results suggest that UL141 uses the same surfaces to interact with TRAIL-R1 and TRAIL-R2 receptor. If true, then the reduced binding affinity for UL141 toward TRAIL-R1 is likely due to individual amino acid differences in that region of TRAIL-R1 that is in contact with UL141. Ultimately, it will be of great interest to determine the structural basis of the viral UL141 engagement of the NK cell activating ligand CD155 and to compare the interactions that occur when UL141 binds to the TRAIL DRs.

Viral glycoprotein UL141 is now known to be required for restricting the cell surface expression of four cellular proteins, including TRAIL-R1, TRAIL-R2, CD155 and CD112. As CD155 was the first identified target of UL141 [16], the increased sensitivity of cells infected with a HCMVΔUL141 mutant to NK-killing was initially ascribed solely to inhibiting DNAM-1/CD226 activation, which also binds CD112 [13]. However, NK cells also express high levels of TRAIL when activated by interferons during viral infection [35], and it is a likely possibility that the potent NK inhibition by UL141 is due to its dual role in modulating multiple effector pathways such as DNAM-1/CD226 NK cell activation as well as TRAIL-mediated killing [12].

Viral manipulation of the immune response is typically achieved by virulence factors, which often imitate the function of a host protein by mimicking its key structural features [36], [37]. One possibility is that a virus first hijacks a host gene(s), and then further evolves/selects those genes for specific functions to target host immune signaling pathways [38]. In this case, virulence factors and host proteins would be derived from the same origin, and differences in the structure and/or function of the viral ortholog would arise by divergent evolution. However, structural mimics can also be generated through convergent evolution [36]. Although differing in evolutionary origin and three-dimensional structure [39], in this case virulence factors evolve to mimic key structural features of cellular proteins. Examples of the latter strategy, which can only be revealed through structural analysis, are fewer than those that can be identified by primary sequence similarity [37], [40], [41]. While UL141 does not display any sequence homology to other proteins in the database, a DALI search ([42], http://ekhidna.biocenter.helsinki.fi/dali_server), identified significant structural conservation with Ig-domain proteins, including T cell receptors, MHC molecules and immunoglobulins (500 proteins with a Z-score of 8.1–9.6 and rmsd of 2.3–4.7 Å aligned over 112 residues in average). A Dali search with only the C-terminal domain (residues 162–246) did not identify structurally related domains. The best hit had a Z-score of 1.2, where similarities with a Z-score lower than 2.0 are spurious. Therefore, the structural conservation is limited to the N-terminal Ig-like domain of UL141 (residues 32–161), while no homology is currently found in the C-terminal domain, indicating this domain adopts a unique structural fold. Interestingly, while the top hit of the DALI search corresponds to a variable TCR β chain (Z = 9.6, rmsd = 3.1 Å over 50% of TCR β chain sequence with total of 119 residues aligned), the second hit was the HCMV protein UL16, an immunoevasin that subverts NKG2D-mediated immune responses by retaining a select group of NKG2D ligands inside the cell [36]. UL16 aligns with 85% of its structure to the Ig-domain of UL141 (Z = 9.4, rmsd = 3.7 Å over 115 residues aligned). However, while the top two structural homologs of UL141 (TCR β and UL16) both bind to MHC-like molecules, UL141 has also evolved to target the TNFRs, illustrating the functional versatility of the Ig-fold.

To our knowledge, the structural and binding data presented here is the first for a viral glycoprotein that directly binds to both a TNFR and an Ig-domain protein. Currently, the only other known example of a TNFR binding to an Ig molecule is HVEM-BTLA, which was recently structurally characterized [33]. HVEM also binds the TNF-family ligand LIGHT. HVEM-BTLA interaction can lead to both inhibition of immune cells through BTLA signaling and activation through HVEM, while LIGHT binding to HVEM is thought to exclusively mediate co-stimulatory signals [43]. Notably, UL144, the HVEM ortholog encoded by HCMV, has evolved to only bind BTLA and not LIGHT, and this has resulted in it being an extremely potent inhibitor of T cell activation [44]. Consequently, both UL144 and UL141 have evolved to target non-canonical interactions of TNFRs with Ig-domain proteins. Our structural analysis has revealed that HCMV has evolved the pleiotropic UL141 as a potent inhibitor of at least two different immune effector pathways, the TRAIL DRs and nectin-like NK cell activating ligands. Our findings provide new insights into the structural basis of the evolutionary dynamic that exists between persistent viruses and host defenses, exemplified by the promiscuous targeting of immune effector pathways by UL141.

In conclusion, our studies on the structure and function of UL141 interacting with its cellular partners reveals valuable insight into the methods employed by this virus to manipulate the human immune system which can occur through non-canonical interactions. Thus, it is extremely important to further explore the nature of these poorly understood interactions, which will likely reveal new modalities that can be exploited for the design of therapeutics.

## Materials and Methods

### Design of expression constructs – UL141, TRAIL-R2

The mature ectodomains of UL141 (amino acids (aa) 30–279 and 30–217, HCMV FIX strain) and TRAIL-R2 (DR5; aa 58–184) were PCR amplified and cloned downstream of the gp67 signal sequence into the baculovirus transfer vector pAcGP67A (BD biosciences) upstream of the Fc domain of human IgG1 (pAc-gp67A-MCS-Thr-Fc; for use in Biacore). A thrombin protease cleavage site (LVPRGS) was also introduced between the individual ectodomain and the Fc fusion protein. In parallel, both UL141 ectodomain constructs were also cloned in pAcGP67A containing only a C-terminal hexa-histidine tag. The UL141 constructs were amplified by polymerase chain reaction (PCR) using human cytomegalovirus (HCMV) cDNA as a template. For amplification of TRAIL-R2, human full-length cDNA was used as a PCR template. A nested PCR protocol with two pairs of primers was used to generate the constructs (Table S2). One pair of primers (hcmvUL141/30for/BamHI and hcmvUL141/217rev/His/EcoRI) generated a DNA product coding for residues 30–217 of UL141 followed by C-terminal hexa-histidine-tag, where forward primer introduced a BamHI restriction site and the reverse primer an EcoRI restriction site with preceding stop codon. Similarly, the second pair of primers (hcmvUL141/30for/BamHI and hcmvUL141/279rev/His/EcoRI) generated a DNA fragment coding for residues 30–279 of UL141. The Fc-fusion expression constructs were generated by amplifying corresponding DNA genes and further ligated into the C-terminal Fc-fusion protein containing baculovirus transfer vector (pAc-gp67A-MCS-Thr-Fc). The following pairs of primers were used in PCR: TRAIL-R2 58–184 gene (huTRAIL-R2-Fc/58for/EcoRI and huTRAIL-R2-Fc/184rev/PstI), UL141 37–247 gene (hcmvUL141-Fc/37for/EcoRI and hcmvUL141-Fc/247rev/PstI) and UL141 37–273 gene (hcmvUL141-Fc/37for/EcoRI and hcmvUL141-Fc/273rev/PstI). The identity and correct sequence of all PCR-amplified constructs was confirmed by sequencing.

### Preparation of recombinant baculoviruses

The baculovirus transfer vector pAcGP67A containing the UL141 or TRAIL-R2 expression construct was amplified in bacteria (*E. coli* DH5α) and maintained under sterile conditions. To increase transfection efficiency, transfection was performed in serum-free media (HyClone SFX-Insect Cell Culture Media, Thermo Scientific) without any antibiotics using Cellfectin reagent (Invitrogen) according to manufacturer's instructions. The transfection complex was formed as follows: 2 µg of recombinant DNA (UL141 or TRAIL-R2 in transfer vector)+0.1 µg of BaculoGold DNA (Invitrogen)+10 µl of Cellfectin Reagent were filled up to 1 ml with media. As a negative control, 20 µl of Cellfectin+1 ml media was mixed. The transfection mixture was vigorously vortexed for 30 sec and incubated at RT for 15 min in the dark. 2×10^6^ healthy-dividing *Spodoptera frugiperda* (SF)9 cells were seeded in T-25 (25 cm^2^) flasks. Culture media was removed and transfection mixture was added drop-wise. Transfection plates were then incubated at RT for 4 hours while rocking back-and-forth every 30 min in dark. After 4 hours, the transfection mixture was replaced with 5 ml fresh media containing antibiotics (mixture of 50 U/ml of penicillin and 50 µg/ml of streptomycin) and plates were incubated at 28°C for 7 days. For the initial screening for positive recombinant UL141 or TRAIL-R2 virus the dilution virus pool method was applied. Positive recombinant virus was selected and then amplified as follows. Cell supernatant containing recombinant virus was collected (1000× g for 10 min) and used for first round of virus amplification. 300 µl of virus with a multiplicity of infection below 1 (MOI<1) was used to infect 2×10^6^ cells in T-25 flask and the flask was then incubated at 28°C. After 5 days, the second virus amplification was performed in T-175 flask to infect 14×10^6^ cells with volume of 1.5 ml of collected virus from the first amplification (MOI<1) in 50 ml of media and incubated for additional 5 days at 28°C. Virus titer was determined by end-point dilution assay (EPDA). Prior to expression, the high titer virus stock was prepared in several T-175 flasks by infection at MOI = 1 of 14×10^6^ cells in total 50 ml volume of media and incubated for 6 days at 28°C. Each flask was then directly used for infection of 2.5×10^7^ cells in total 1 L volume of media (MOI between 3 to 5) and incubated for 72 to 84 h at 28°C as a suspension culture (at 138 rpm).

### Expression of seleno-methionine labeled UL141–TRAIL-R2–Fc complex

Recombinant virus stock containing both UL141 and TRAIL-R2–Fc virus particles was prepared similar to the individual virus stocks (see above). To achieve equal protein synthesis via baculovirus mediated co-expression in Sf9, we prepared the transfection mixture under the following condition: 2 µg of UL141 recombinant DNA+2 µg of TRAIL-R2 Fc-fusion recombinant DNA (both in separate transfer vectors)+0.5 µg of BaculoGold DNA (Invitrogen)+20 µl of Cellfectin Reagent, filled up to 1 ml with media and as a control, 20 µl of Cellfectin+1 ml media was mixed. The first round of virus amplification was done by infecting the Sf9 cells, which were previously adapted for vital growth in ESF-921 protein-free media (Expression systems, Inc.), with heterologous virus from a 7-day transfection at 28°C. Similarly, the second virus amplification and the high titer virus stocks were prepared in several T-175 flasks by infection at MOI = 1 of 14×10^6^ cells in total 50 ml volume of ESF-921 media and incubated for 6 days at 28°C. Each flask was then directly used for infection of 2.5×10^7^ cells in total 1 L volume of ESF-921 methionine-rich media (MOI between 3 to 5) and incubated for 16 hours at 28°C as a suspension culture (at 138 rpm). To achieve depletion of methionine from intracellular pools, we collected cells at 300 g for 15 min at RT and resuspended in ESF-921 methionine-free media with antibiotics (50 µg/mL gentamycin). Subsequently, seleno-methionine (50 mg/L) was added, to the suspension culture at 28°C. The critical point of seleno-methionine addition is within the first 16–20 hours following viral infection, as the protein expressing begins at that time. Expression of seleno-methionine labeled UL141–TRAIL-R2 Fc-fusion protein complex was continued for 48–96 hours post-infection (total time of expression 3.5 days at 28°C). The culture media containing the seleno-methionine labeled protein complex was separated from cells by centrifugation (1000 g for 10 min) and debris was removed by additional centrifugation at 5500 g for 10 min at 4°C.

### Expression of UL141 Fc and CD155 Fc- and TRAIL-R1 Fc-fusion proteins

Fc-fusion proteins were produced in baculovirus mediated insect cell expression system as well as in mammalian 293T cells. For 293T cells, DNA was prepared using the Endofree Plasmid Maxi kit (Qiagen, Valencia, CA, USA) and maintained under the sterile condition. The confluent 293T cells were passaged in T-175 flasks in D10 media and incubated at 37°C with 5% CO_2_. As a detaching component 0.05% trypsin-EDTA solution was used to further maintain the cells. 293T cells were transfected by standard calcium phosphate transfection method and subsequently maintained for 72 h. The transfection mixture containing 100 µl of 2.5 M CaCl_2_, 22 µg DNA filled up to 1 ml with sterile water (calculation for one T-175 plate) was bubbled into 1 ml of 2× HeBS buffer (containing phosphate) and drop-wise transferred to seeded 293T cell in T-175 flask containing 25 ml D10 medium. After one day of transfection the media was changed to 30 ml CellGro media containing antibiotics and L-glutamine. After 48 hours of expression, the media was changed to fresh and supernatant was collected for harvesting, while rest of the cells in fresh media continues for next 24 hours expression. UL141 Fc protein was purified from cell culture supernatant using a HiTrap Protein A HP column (Amersham Biosciences, Piscataway, NJ, USA), while CD155-Fc and TRAIL-R1 Fc were used directly from culture supernatant for SPR studies (see below).

### HEK293T cell culture

HEK293T cells were grown in Dulbeccos's modified medium (DMEM) supplemented with 10% (v/v) fetal calf serum (FCS), 2 mM L-glutamine and 100 units/ml of penicillin, 100 µg/ml of streptomycin (all together are components of D10 medium). Transfected 293T cells were further maintained in CellGro serum-free, protein-free media (CellGro, Mediatech).

### Western blots

Fc-fusion and His-tagged proteins were run on SDS gradient polyacrylamide gels and transferred to nitrocellulose membranes. Blots were probed with anti human IgG-HRP conjugate for UL141-Fc, CD155-Fc and TRAIL-R2-Fc (BioRad), or with mouse anti-penta-His conjugate and anti-mouse IgG HRP conjugate antibodies (Sigma).

### Purification of UL141–TRAIL-R2 complex (SeMet-labeled and native)

The extracellular domains of TRAIL-R2 and UL141 were cloned into transfer vector pAcGP67A engineered with C-terminal Fc-fusion tag in case of TRAIL-R2 and His-tag for UL141 construct. The proteins were co-expressed via the baculovirus expression system as a non-covalent protein complex, while a cleavage site for thrombin protease was introduced between TRAIL-R2 and the Fc portion of human IgG1. After three days of expression in insect cell media at 28°C, Sf9 cells and debris was removed from the protein containing culture supernatant by centrifugation. The supernatant was concentrated to 500 ml while the buffer was exchanged against 1× PBS by tangential flow-through filtration using 10 kDa molecular weight cut-off membranes (Millipore filtration device, Pelicon-2). Briefly, the UL141-TRAIL-R2-Fc complex was purified by affinity chromatography using Protein A (HiTrap Protein A), followed by Ni^2+^-affinity chromatography using HisTrap (both GE Healthcare), to purify the protein complex, rather than the individual components (Figure S6 and Figure S7). Next, the UL141-TRAIL-R2-Fc containing fractions were pooled and dialysed at 4°C against 10 mM TRIS pH 8.0 buffer for subsequent purification by anion-exchange chromatography using MonoQ (GE Healthcare) and a 0–1 M sodium chloride gradient (Figure S6b). The UL141–TRAIL-R2 complex was further released from the Fc fusion tag by thrombin (Sigma) digestion at RT for 2 h, using 1 U of thrombin per mg of protein complex. Free Fc protein as well as uncleaved complex was further removed by affinity chromatography using Protein A resin (Figure S6c). During final purification by size exclusion chromatography (SEC) using Superdex S200 (GE Healthcare), the UL141–TRAIL-R2 complex eluted as a roughly 90 kDa peak consistent with one UL141 dimer binding two TRAIL-R2 monomers. The protein complex migrated as two major bands (38 and 19 kDa) on both reducing and non-reducing SDS gels (Figure S7).

### Crystallization of UL141–TRAIL-R2 protein complex (native and SeMet-labeled)

The UL141–TRAIL-R2 containing fractions for both native and selenomethionine labeled protein were pooled and concentrated to final concentrations of 7.3 mg/ml (native) and 8.3 mg/ml (labeled) in 50 mM HEPES, 150 mM NaCl, pH 7.5. Initial crystallization trials were carried out by robotic crystallization (Phoenix, Art Robbins Instruments) using the sitting drop vapor diffusion method at room temperature as well as 4°C. Over 700 conditions were screened using several different commercial crystallization screens (Wizard I, II, III; PEG-ion 1, 2; JSCG I-IV and Core; Hampton Research Additive Screen) to find several initial crystallization hits for UL141-TRAIL-R2 native and one condition for labeled complex. Three-dimensional native and derivative crystals of the UL141–TRAIL-R2 protein complex were grown at 22°C in the presence of high pH buffer (CHES 9.5 and bicine 9.0, respectively) and 20% (w/v) polyethylene glycol (8000 and 6000, respectively). The derivative condition also includes 0.2 M calcium chloride and 5–10% glycerol as an additive. These crystals were further optimized by macro- and micro-seeding techniques, as well as by crystallization under oil to improve diffraction quality. Crystallization under oil and crystallization with glycerol were the most successful optimization. A 5–8 µl drop containing a 1∶1 mixture of Silicon and Paraffin Oil (Hampton Research), also known as Al's Oil, was placed as sitting drop. Next, the protein and precipitant (see above) was mixed 1∶1 and pipetted under the oil. Reservoir was filled up with 1 ml of precipitant solution. Crystals were grown slowly over several days to maximal dimensions of approximately 1000×30×40 µm.

### Data collection and processing – UL141–TRAIL-R2 native and derivative data

Crystals were cryo-protected in well solution containing 25% glycerol and then flash-cooled in liquid nitrogen for data collection at 100 K. X-ray diffraction data were collected on CCD type detector (model ADSC Quantum 315r) from the six best diffracting crystals at the Stanford Synchrotron Radiation Lightsource (SSRL) beamline 7-1 after testing crystals by excitation scan at Se-K edge for Se incorporation. The wavelength used for data collection was at the peak of Se f″ (0.9795 Å, 12667 keV). The inverse-beam mode of data collection was used with 7 sec exposure time (the crystal was rotated 180° every 10 frames to measure Friedel mates). To better resolve the reflections corresponding to the long axis, the crystals were aligned in the loop with the long axis roughly parallel to the rotational spindle axis. In addition, a long sample-to-detector distance (300 mm) and an oscillation of 0.5° were used to reduce overlaps. The strategy function in iMosflm [45] was used to reduce overlap as well as to maximize data completeness. Five of the six diffraction data sets were selected for analysis. The parameter for collection of the native UL141–TRAIL-R2 dataset at direct beam mode are as follows: crystal-to-detector distance (350 mm), exposure time for 10 sec and oscillation increment was 1°. Both data were indexed and integrated by iMosflm. The crystals of SeMet UL141–TRAIL-R2 belong to space group P2_1_2_1_2_1_, with unit-cell parameters a = 67.9 Å, b = 97.0 Å and c = 141.4. Å and native UL141–TRAIL-R2 with a = 67.7 Å, b = 97.7 Å, c = 141.3 Å.

### Multi-crystal data reduction - UL141–TRAIL-R2 derivative

Each single-crystal data set was indexed and integrated by iMosflm [45]. The CCP4 program (Collaborative Computational Project, Number 4) SCALA [46] was used for data scaling and merging with secondary beam correction and rotational restraints for scale and B factors. The ‘anomalous’ option in SCALA was turned on to allow the separation of Friedel mates in the merged data. For scaling, Friedel mates were not treated separately. A multicrystal dataset was produced by merging the five individual anomalous datasets in SCALA. We generated different sets of multi-crystal data, including and excluding data from crystal C6. The C6 data proved to have appreciably stronger anomalous signal than the others and was sufficient for phasing. Data collection statistics for the native data, the C6 data as well as multi-crystal merged data are presented in Table 2. The strategy for multi-crystal data reduction was adapted from [47].

### Substructure determination and phasing - UL141–TRAIL-R2

Selenium-substructure determinations were performed with the SHELXD program package [48]. A resolution cutoff at 4.5 Å and an *E*_min_ cutoff at 1.4 were initially used to find Se substructures with SHELXD. Trials were made for each data set and for various merged data sets. For each case, 500 attempts were made to find the expected 20 Se sites. For those single-crystal and multi-crystal data sets that did not yield successful Se-substructure determinations using SHELXD, Se substructures were obtained by running Phaser [49] in its MR-SAD mode with addition of phases from the model (PDB coordinates for TRAIL-R2 (1D4V) and incomplete homology model of UL141-Ig-domain, PSI-Protein Model Portal; query No. Q6RJQ3). The model was only used for Se-substructure determination (for weak data only) and was excluded from the subsequent SAD phasing. For all cases, initial SAD phases were calculated by Phaser. For two selected cases (C6 alone and multi-crystal data including C6), these initial phases were subjected to automatic density modification with solvent flattening and histogram matching as implemented in the CCP4 program DM and DM-Multi [50]. An estimated solvent content of 51% was used for the density modification procedure. Map correlation coefficients (map CCs) and mean phase errors were calculated to compare the resulting experimental phases with model phases. In order to improve density, we used Uniqueify to generate Free R value and FFT to generate anomalous density map. Automated model builder ARP/wARP generated polyalanine model and Buccaneer (all of CCP4 package) together with AutoRickshaw structure solving module [51] were used to extend this model by searching for TRAIL-R2 (from PDB 1D4V). The first interpretable model of UL141–TRAIL-R2 heterodimer was then rebuilt into σA-weighted 2Fo–Fc and Fo–Fc difference electron density maps using the program COOT [52]. Final steps included the TLS (Translation-Libration-Screw) procedure [53] in REFMAC5 [54] with three TLS domains (residues 80–180 of TRAIL-R2, 34–165 and 176–198 of UL141). The UL141–TRAIL-R2 structure was refined to 2.1 Å using multi-crystal data with a final Rfree of 27.4%. The quality of the model was examined with the program Molprobity [55].

### PDB accession numbers

Coordinates and structure factors for the UL141–TRAIL-R2 structure have been deposited in the Protein Data Bank under accession code 4I9X.

### Surface plasmon resonance

After purification, the proteins were concentrated with an Amicon Centrifugal Filter Unit (Millipore, Ultracell-30K or 10K) and the buffer was exchanged against 10 mM HEPES pH 7.4, 150 mM sodium chloride and 3 mM EDTA (as Biacore running buffer). The proteins were diluted in Biacore running buffer containing 0.005% Tween 20 to appropriate concentration prior to loading. An anti-human Fc capture antibody was immobilized on a CM5 sensor chip (GE Healthcare) by amine coupling. Approximately 500–1000 response units (RU) of TRAIL-R2–Fc, TRAIL-R1–Fc, UL141–Fc and CD155–Fc were captured on sensor chip. TRAIL-R1 Fc, CD155 Fc and TRAIL-R2 Fc mutant proteins were captured on the sensor chip directly from the filtered culture supernatant. The serial dilutions of UL141 protein (0–0.5 µM), TRAIL-R2 receptor (0–1 µM), UL141–TRAIL-R2 protein complex (0–10 µM) were prepared in running buffer. The analytes were then injected in duplicates for 5 to 10 min association, while dissociation was conducted over 30 min. After each cycle, the chip was regenerated with a 30 sec injection of 2 M MgCl_2_ at 15 µl/min and freshly coated with ligand (Fc-fusion protein). Experiments were carried out at 18°C with a flow rate of 10 to 30 µl/min and performed in several repeats, each time with a different stock preparation (except for the experiment with TRAIL-R1, this was performed only once). As a negative control for unspecific binding, human LTβR–Fc (Lymphotoxin β receptor from TNFR family) was immobilized on the first flow-channel (it is know that UL141, TRAIL-R2, -R1 nor CD155 do not bind to LTβR). Kinetic parameters were calculated after subtracting the response to the negative control (LTβR–Fc) and next the buffer only control as a background, using a simple Langmuir 1∶1 model in the BIA evaluation software version 4.1

### Glycan modeling

Three potential N-linked glycosylation sites were identified in the UL141 ectodomain. All of the possible asparagine residues (Asn117 in chain A, Asn132 in chain B, and Asn147 in both chains) carry one or two NAG (N-acetylglucosamine) residues that are clearly defined by electron density. While extra density is present also at the Asn117 (in chain B) and as well as Asn132 (in chain A), this density is not well defined, and no NAG was build in this location in crystal structure, but we incorporated this sites in modeling as they are occupied in adjacent UL141 subunit. We used energy-minimized PDB coordinates for basic mannose containing N-linked carbohydrates (GlcNAG_2_-Man_2_) to visualize the surface accessibility on UL141.

### Generation of human TRAIL-R2–Fc mutants

Human TRAIL-R2 Fc-fusion mutants (Table S3) were generated by site-directed mutagenesis using Quick Change II Multi-site Mutagenesis Kit (Stratagene, La Jolla, CA, USA). Single mutations were incorporated using the Quick Change II Site-Directed Mutagenesis Kit (Stratagene, Agilent Technologies). Mutated constructs were purified with the Qiagen Miniprep Kit (Qiagen) and the presence of the mutation confirmed by sequencing. All mutants of human TRAIL-R2–Fc were expressed in Sf9 insect cells and the culture supernatant was used for SPR studies.

## Supporting Information

Figure S1 **Size-exclusion profiles of purified proteins.** Size-exclusion elution profiles of UL141 dimer (**a**), TRAIL-R2 monomer (**b**), TRAIL-R1-Fc dimer (**c**), and TRAIL-R2-Fc dimer (**d**). The purified proteins (shaded areas) elute as mono-disperse peaks. Calibration curve with molecular weight of marker proteins in kDa is shown in grey.
(TIF)

Figure S2 **Sequential binding experiment assessed by SPR.** Human CD155-Fc was immobilized on flow channel 2 (Fc-2, bold line) of a CM5 sensorchip, while Fc-1 served as negative control (dashed line). UL141 was passed over both channels (Fc-1 and Fc-2) and allowed to bind to CD155-Fc, followed by injection of TRAIL-R2, for which additional binding to UL141 was observed. Protein injections are indicated by arrows.
(TIF)

Figure S3 **Sequence alignment of various TNF ligands with UL141 (a) and TNFSFR (b).** Residues that are conserved throughout the TNF family are shaded in blue according percentage identity (dark blue for identical residue). Residues that form a particular binding patch in the UL141–TRAIL-R2 structure are boxed using the colors of Figure 4. (**a**) **TNF ligands:** TNFSF1/TNFβ/LTα (1-205), TNFSF2/TNFα (1-233), TNFSF6/FasL/CD96L (1-281), TNFSF10/TRAIL/Apo2L (1-281) and TNFSF11/RANKL/TRANCE/OpgL (1-317). (**b**) **TNF receptors:** TNFRSF10A/TRAIL-R1/DR4 (1-468), TNFRSF10B/TRAIL-R2/DR5 (1-440), TNFRSF10C/TRAIL-R3/DcR1 (1-259), TNFRSF10D/TRAIL-R4/DcR2 (1-386), TNFRSF11A/RANK (1-616), TNFRSF11B/OPG/OCIF (1-401), TNFRSF1A/TNFR1 (1-455), TNFRSF1B/TNFR2 (1-461) and TNFRSF6/Fas/APT1 (1-335).
(TIF)

Figure S4 **Membrane embedding model.** Comparison of both TRAIL (cyan) and UL141 (yellow) bound to TRAIL-R2 on cellular membranes. Arrows highlight approximate distances between C-termini of TRAIL-R2 (in blue), C-termini of UL141 (in red) and N-termini of TRAIL ligand (in red) embedded in the membrane (or as soluble TRAIL trimers, not depicted). As indicated, TRAIL-R2 lacks an additional 28 residues (+28 aa) before entering the membrane via the TM domain, while UL141 lacks 34 residues (+34 aa). The C-termini of TRAIL-R2 would be significantly more separated (>90 Å) than in the TRAIL–TRAIL-R2 complex (∼50 Å) indicating a possible mechanism by which UL141 prevents TRAIL-R2 mediated signaling, in addition to the ER retention of TRAIL-R2 by UL141.
(TIF)

Figure S5 **Representative SPR traces and residual plots for binding data reported in** **Table 1** **.** Kinetics binding data for UL141-Fc vs. TRAIL-R2 (**a**), CD155-Fc vs. UL141 (**d**) and CD155-Fc vs. UL141–TRAIL-R2 (**e**) including residual plot and statistics. For details, see Table 1.
(TIF)

Figure S6 **Purification of UL141–TRAIL-R2 Fc–fusion protein complex.** Purification of seleno-methionine (SeMet) labeled UL141–TRAIL-R2 protein complex from *Spodoptera Frugiperda* (Sf9) insect cells. (**a**) Affinity chromatography by His-tag capturing Ni-NTA agarose column (Hi-TRAP 1 ml column, GE Healthcare) performed by linear step gradient of Imidazole. (**b**) Anion exchange chromatography (Mono Q 1 ml column, GE Healthcare) performed by gradient of sodium chloride. (**c**) Human Fc-protein affinity chromatography using Protein A (HiTrap 1 ml column, GE Healthcare) after Thrombin cleavage. (**d**) Size exclusion chromatography (Superdex S200 10/300 column, GE Healthcare) elution profile of SeMet-UL141-TRAIL-R2 protein complex. Shaded areas represent SeMet-UL141-TRAIL-R2-containing fractions. Calibration curve is shown in grey with MW markers indicated in kDa. For experimental details see methods.
(TIF)

Figure S7 **SDS-PAGE of the UL141–TRAIL-R2 Fc–fusion protein complex.** Gradient 4–20% SDS-PAGE of freshly purified samples of UL141-TRAIL-R2 Fc-fusion protein complex under reducing (R) and non-reducing (NR) condition. Lanes 3 and 4 are samples treated by one unit (1 U) of Thrombin (Thr) per mg of protein. MW of maker proteins indicated in kDa.
(TIF)

Table S1 **Determination of the binding contribution accessed by surface plasmon resonance of a specific residue by alanine scanning on TRAIL-R2.**
(PDF)

Table S2 **PCR cloning primers for UL141 and TRAIL-R2 expression constructs.**
(PDF)

Table S3 **List of multi-site mutation primers.**
(PDF)
