# Supplementary figures and images for: Structure of Human Cytomegalovirus UL141 Binding to TRAIL-R2 Reveals Novel, Non-canonical Death Receptor Interactions
Source: PLoS Pathog. 2013 Mar 21;9(3):e1003224. doi: 10.1371/journal.ppat.1003224 (PMC3605307; doi:10.1371/journal.ppat.1003224)

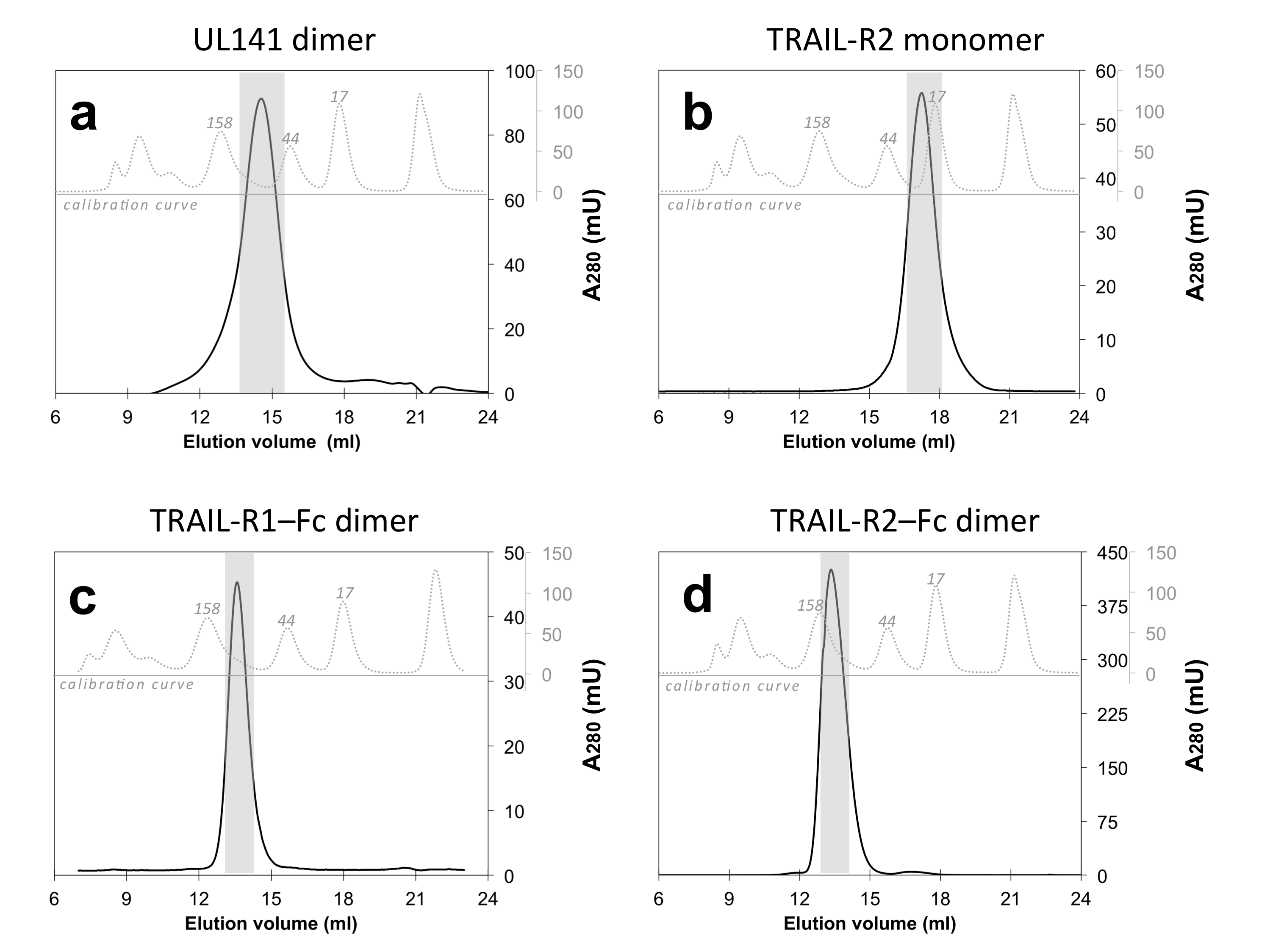

Supplement: Figure S1 — Size-exclusion profiles of purified proteins. Size-exclusion elution profiles of UL141 dimer (a), TRAIL-R2 monomer (b), TRAIL-R1-Fc dimer (c), and TRAIL-R2-Fc dimer (d). The purified proteins (shaded areas) elute as mono-disperse peaks. Calibration curve with molecular weight of marker proteins in kDa is shown in grey. (TIF) [file ppat.1003224.s001.tif]

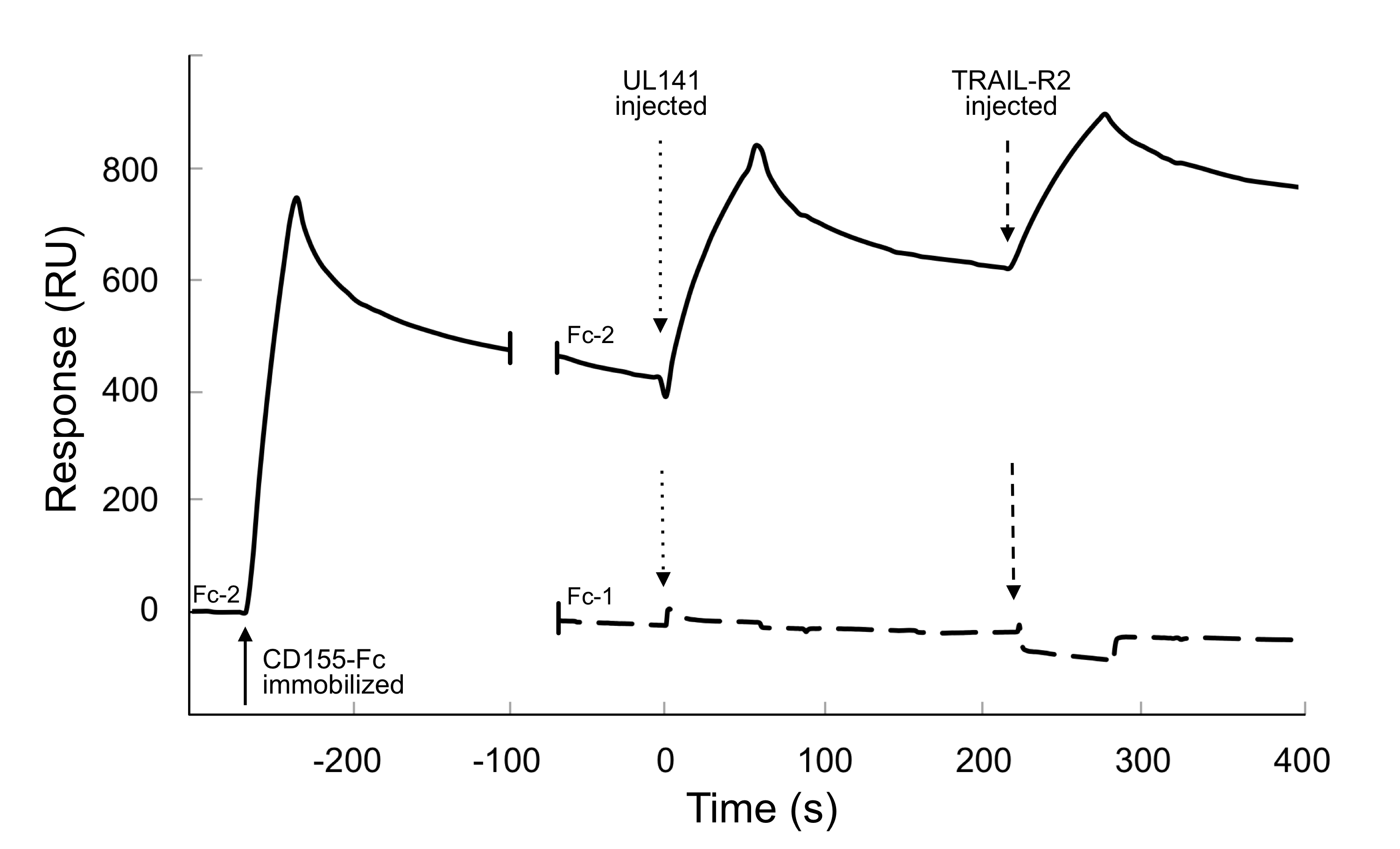

Supplement: Figure S2 — Sequential binding experiment assessed by SPR. Human CD155-Fc was immobilized on flow channel 2 (Fc-2, bold line) of a CM5 sensorchip, while Fc-1 served as negative control (dashed line). UL141 was passed over both channels (Fc-1 and Fc-2) and allowed to bind to CD155-Fc, followed by injection of TRAIL-R2, for which additional binding to UL141 was observed. Protein injections are indicated by arrows. (TIF) [file ppat.1003224.s002.tif]

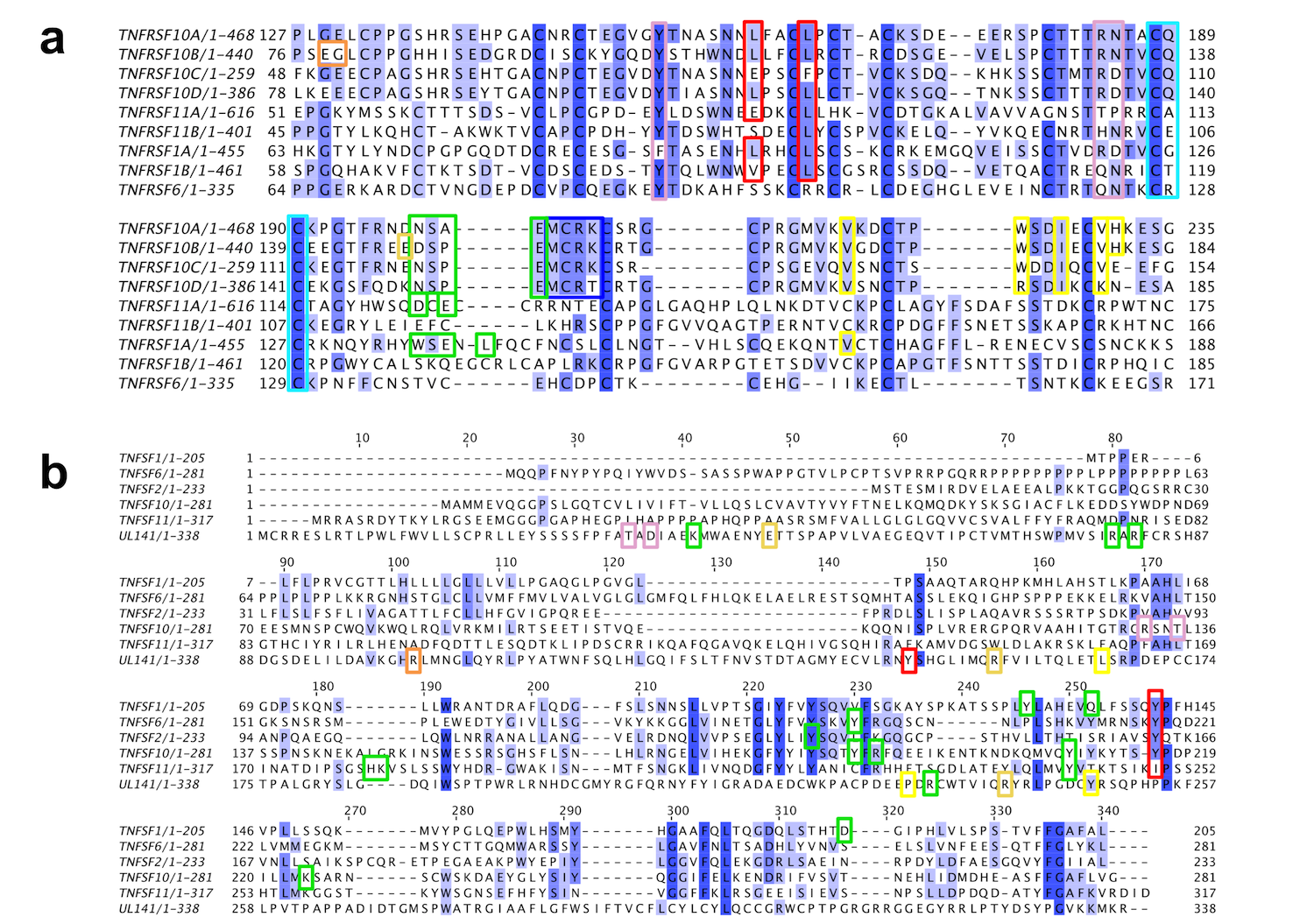

Supplement: Figure S3 — Sequence alignment of various TNF ligands with UL141 (a) and TNFSFR (b). Residues that are conserved throughout the TNF family are shaded in blue according percentage identity (dark blue for identical residue). Residues that form a particular binding patch in the UL141–TRAIL-R2 structure are boxed using the colors of Figure 4. (a) TNF ligands: TNFSF1/TNFβ/LTα (1-205), TNFSF2/TNFα (1-233), TNFSF6/FasL/CD96L (1-281), TNFSF10/TRAIL/Apo2L (1-281) and TNFSF11/RANKL/TRANCE/OpgL (1-317). (b) TNF receptors: TNFRSF10A/TRAIL-R1/DR4 (1-468), TNFRSF10B/TRAIL-R2/DR5 (1-440), TNFRSF10C/TRAIL-R3/DcR1 (1-259), TNFRSF10D/TRAIL-R4/DcR2 (1-386), TNFRSF11A/RANK (1-616), TNFRSF11B/OPG/OCIF (1-401), TNFRSF1A/TNFR1 (1-455), TNFRSF1B/TNFR2 (1-461) and TNFRSF6/Fas/APT1 (1-335). (TIF) [file ppat.1003224.s003.tif]

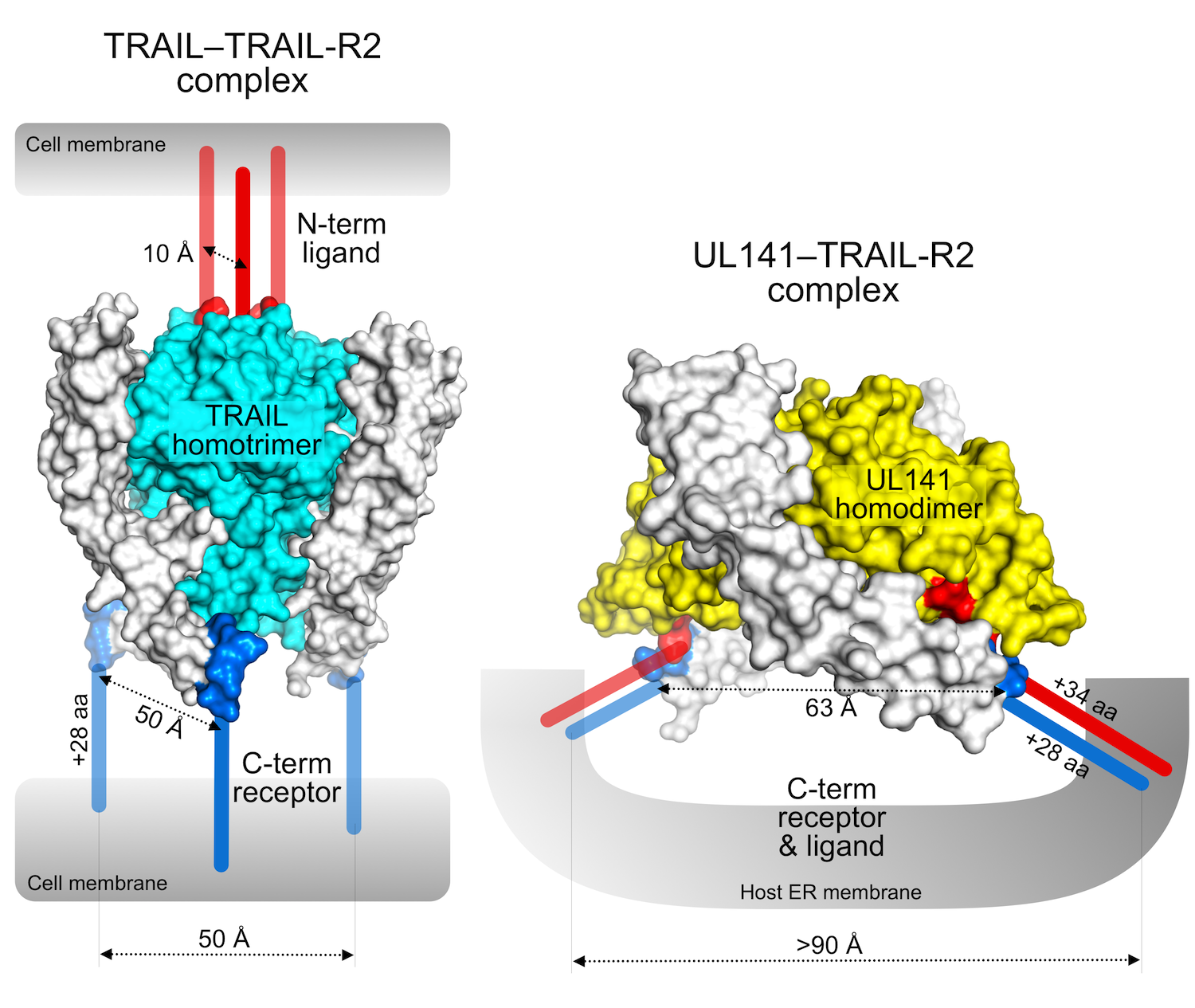

Supplement: Figure S4 — Membrane embedding model. Comparison of both TRAIL (cyan) and UL141 (yellow) bound to TRAIL-R2 on cellular membranes. Arrows highlight approximate distances between C-termini of TRAIL-R2 (in blue), C-termini of UL141 (in red) and N-termini of TRAIL ligand (in red) embedded in the membrane (or as soluble TRAIL trimers, not depicted). As indicated, TRAIL-R2 lacks an additional 28 residues (+28 aa) before entering the membrane via the TM domain, while UL141 lacks 34 residues (+34 aa). The C-termini of TRAIL-R2 would be significantly more separated (>90 Å) than in the TRAIL–TRAIL-R2 complex (∼50 Å) indicating a possible mechanism by which UL141 prevents TRAIL-R2 mediated signaling, in addition to the ER retention of TRAIL-R2 by UL141. (TIF) [file ppat.1003224.s004.tif]

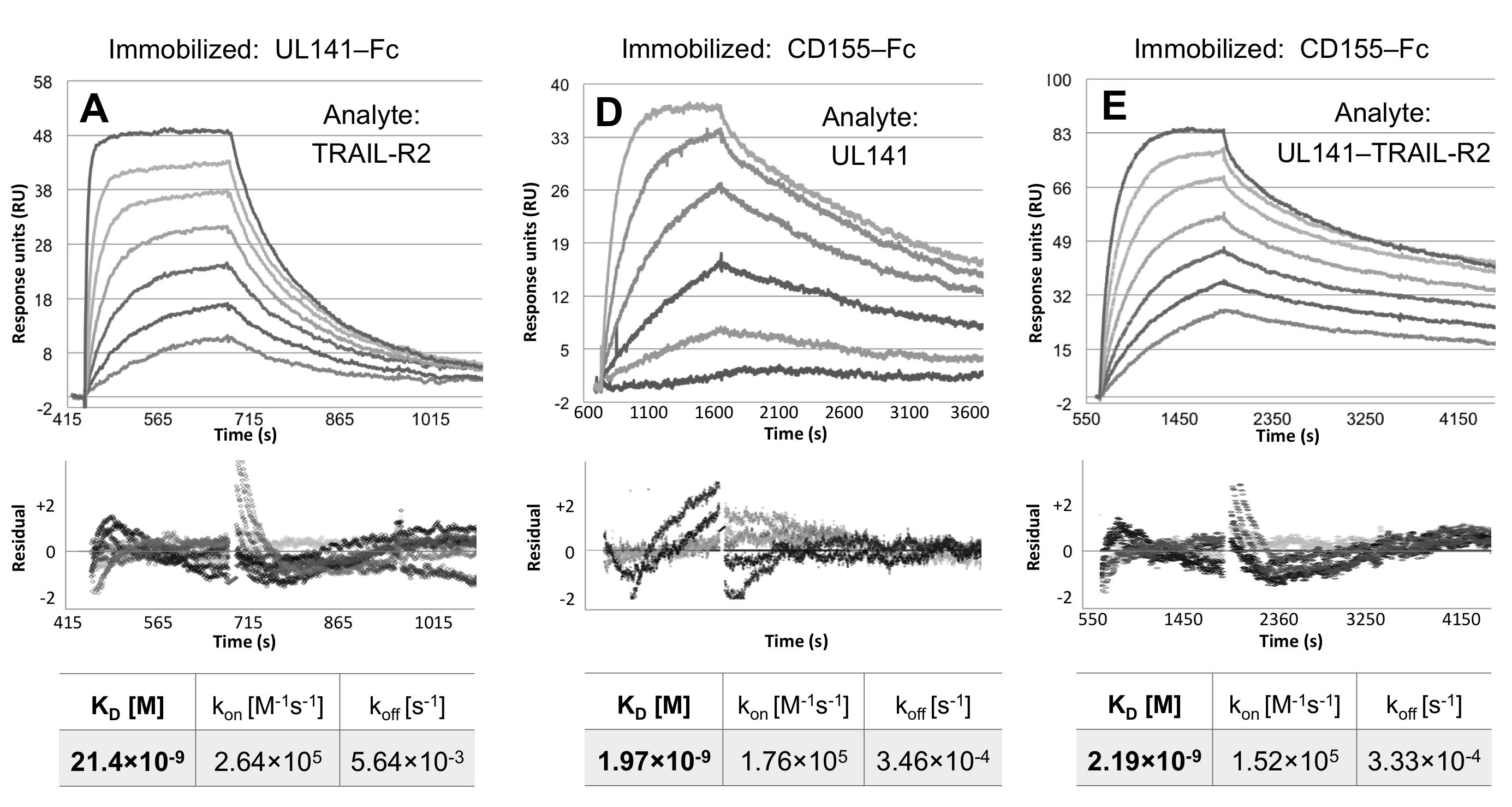

Supplement: Figure S5 — Representative SPR traces and residual plots for binding data reported in Table 1 . Kinetics binding data for UL141-Fc vs. TRAIL-R2 (a), CD155-Fc vs. UL141 (d) and CD155-Fc vs. UL141–TRAIL-R2 (e) including residual plot and statistics. For details, see Table 1. (TIF) [file ppat.1003224.s005.tif]

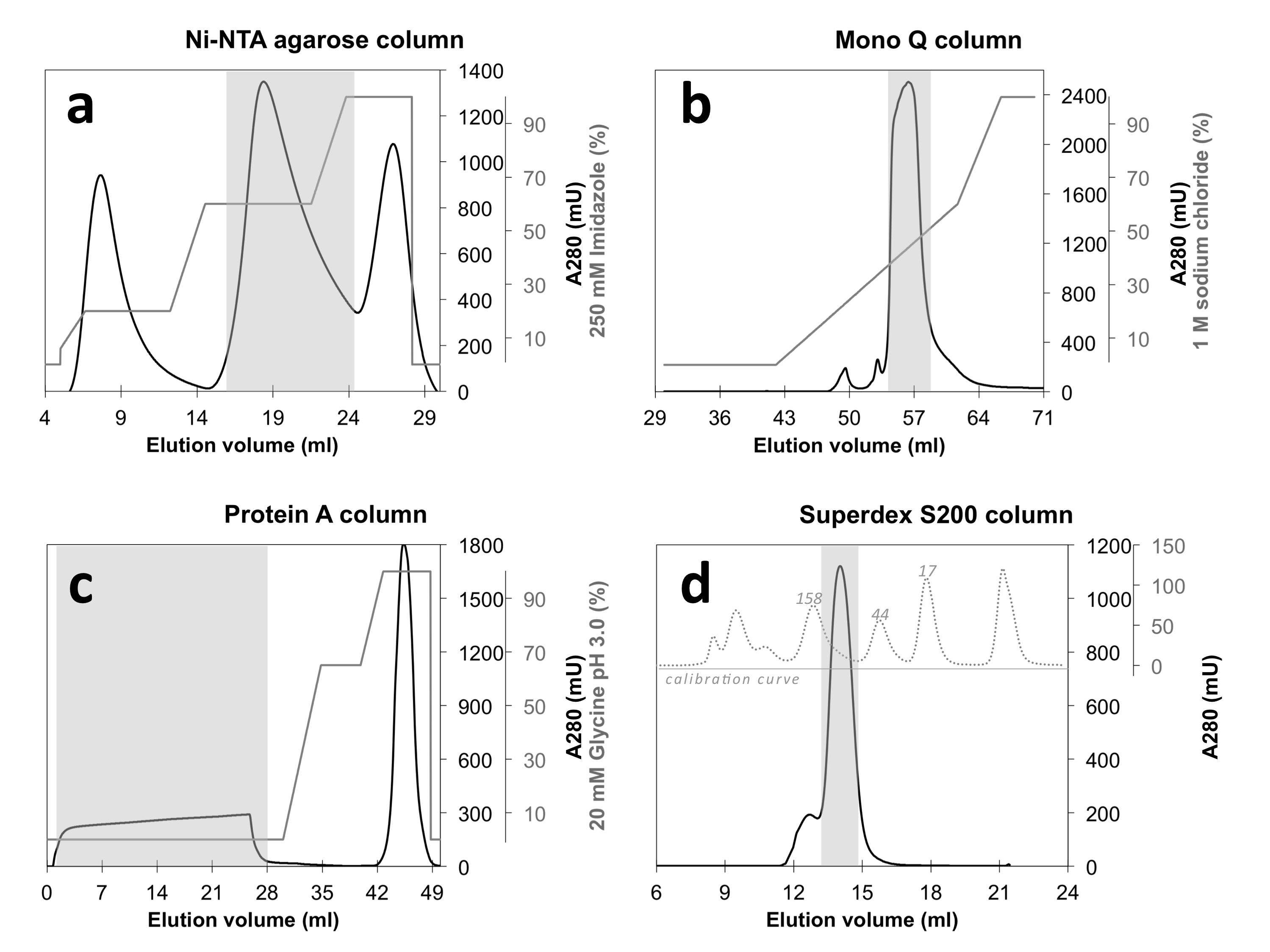

Supplement: Figure S6 — Purification of UL141–TRAIL-R2 Fc–fusion protein complex. Purification of seleno-methionine (SeMet) labeled UL141–TRAIL-R2 protein complex from Spodoptera Frugiperda (Sf9) insect cells. (a) Affinity chromatography by His-tag capturing Ni-NTA agarose column (Hi-TRAP 1 ml column, GE Healthcare) performed by linear step gradient of Imidazole. (b) Anion exchange chromatography (Mono Q 1 ml column, GE Healthcare) performed by gradient of sodium chloride. (c) Human Fc-protein affinity chromatography using Protein A (HiTrap 1 ml column, GE Healthcare) after Thrombin cleavage. (d) Size exclusion chromatography (Superdex S200 10/300 column, GE Healthcare) elution profile of SeMet-UL141-TRAIL-R2 protein complex. Shaded areas represent SeMet-UL141-TRAIL-R2-containing fractions. Calibration curve is shown in grey with MW markers indicated in kDa. For experimental details see methods. (TIF) [file ppat.1003224.s006.tif]

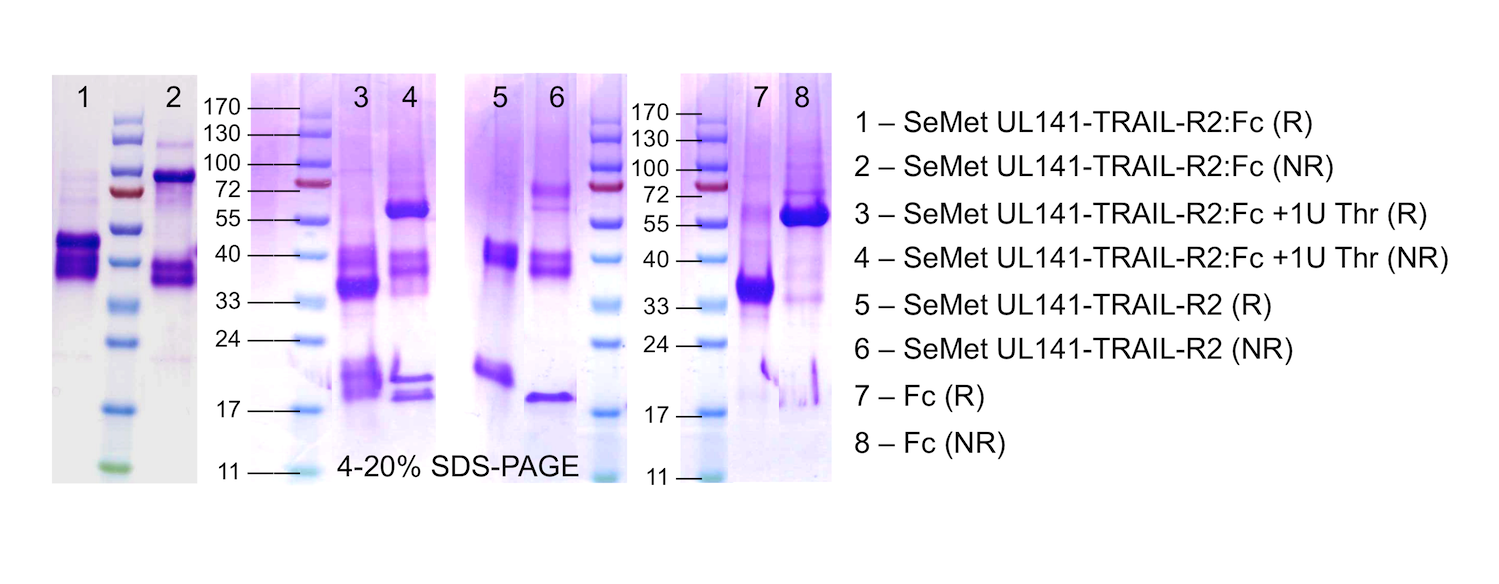

Supplement: Figure S7 — SDS-PAGE of the UL141–TRAIL-R2 Fc–fusion protein complex. Gradient 4–20% SDS-PAGE of freshly purified samples of UL141-TRAIL-R2 Fc-fusion protein complex under reducing (R) and non-reducing (NR) condition. Lanes 3 and 4 are samples treated by one unit (1 U) of Thrombin (Thr) per mg of protein. MW of maker proteins indicated in kDa. (TIF) [file ppat.1003224.s007.tif]
