# Supplementary material for: Structure of Human Cytomegalovirus UL141 Binding to TRAIL-R2 Reveals Novel, Non-canonical Death Receptor Interactions
Source: PLoS Pathog. 2013 Mar 21;9(3):e1003224. doi: 10.1371/journal.ppat.1003224 (PMC3605307; doi:10.1371/journal.ppat.1003224)
Supplement: Table S1 — Determination of the binding contribution accessed by surface plasmon resonance of a specific residue by alanine scanning on TRAIL-R2. (PDF) [file ppat.1003224.s008.pdf]

Table S1. Determination of the binding contribution accessed by surface plasmon resonance of a specific residue by alanine scanning on TRAIL-R2.

Patch No.	Alanine mutation	Binding to TRAIL	Binding to UL141	K_D [nM]	K_{on} [$M^{-1}s^{-1}$]	K_{off} [s^{-1}]	R_{max}	χ^2
	WT	—	6 nM	5.95	1.21×10^4	7.21×10^{-5}	210	1.5
		4 nM	—	3.96	0.23×10^4	9.11×10^{-6}	115	2.5
3	P150	—	YES	8.32	4.70×10^4	3.91×10^{-4}	101	1.5
		YES	—	4.29	0.21×10^4	9.02×10^{-6}	130	3.1
	E151	NO	NO	*n.d.**	*n.d.**	*n.d.**	*n.d.**	*n.d.**
3U	D148	—	10-fold lower	55.32	2.35×10^4	1.30×10^{-3}	100	2.7
		YES	—	4.48	0.80×10^4	3.58×10^{-5}	122	2.5
5	L110/L114	—	10-fold lower	42.67	1.50×10^4	6.40×10^{-4}	68	1.2
		NO	—	*n.d.**	*n.d.**	*n.d.**	*n.d.**	*n.d.**
	F112	—	100-fold lower	630.28	8.98×10^3	5.66×10^{-3}	248	1.8
		YES	—	3.94	0.25×10^4	9.86×10^{-6}	150	3.8
3T	M152	—	YES	7.49	5.10×10^4	3.82×10^{-4}	400	1.3
		50-fold lower	—	202.38	3.35×10^3	6.78×10^{-4}	118	2.2
	R154	—	YES	6.26	3.88×10^4	2.43×10^{-4}	320	1.9
		10-fold lower	—	45.70	2.56×10^3	1.17×10^{-4}	109	2.7
	K155	—	YES	7.74	4.91×10^4	3.80×10^{-4}	352	1.2
		10-fold lower	—	39.66	3.48×10^3	1.38×10^{-4}	120	2.9
	M152/R154/K155	—	YES	7.39	3.65×10^4	2.70×10^{-4}	69	1.1
		NO	—	*n.d.**	*n.d.**	*n.d.**	*n.d.**	*n.d.**
4	Y103/R133	NO	NO	*n.d.**	*n.d.**	*n.d.**	*n.d.**	*n.d.**
	Y103/N134	NO	NO	*n.d.**	*n.d.**	*n.d.**	*n.d.**	*n.d.**
1	V167	—	2.5-fold lower	15.48	3.10×10^4	4.80×10^{-4}	74	1.1
		YES	—	4.35	0.22×10^4	9.57×10^{-6}	400	4.1
1-2	V167/W173/V179	—	NO	*n.d.**	*n.d.**	*n.d.**	*n.d.**	*n.d.**
		YES	—	4.15	0.26×10^4	1.08×10^{-5}	35	2.8
6	E78/D109	—	10-fold lower	60.4	2.5×10^4	1.51×10^{-3}	35	1.9
		YES	—	3.92	0.23×10^4	9.01×10^{-6}	442	2.9

(*) *n.d.* – Binding was not detected.
