# Supplementary material for: Structure of Human Cytomegalovirus UL141 Binding to TRAIL-R2 Reveals Novel, Non-canonical Death Receptor Interactions
Source: PLoS Pathog. 2013 Mar 21;9(3):e1003224. doi: 10.1371/journal.ppat.1003224 (PMC3605307; doi:10.1371/journal.ppat.1003224)
Supplement: Table S2 — PCR cloning primers for UL141 and TRAIL-R2 expression constructs. (PDF) [file ppat.1003224.s009.pdf]

Table S2. PCR cloning primers for UL141 and TR2 expression constructs.

hcmvUL141/30for/BamHI
5'- CCGGGATCCCTCGTTCCCCTTCGCCACCG -3'
hcmvUL141/217rev/His/EcoRI (short)
5'- CCGGAATTCTCAGTGATGGTGATGGTGATGGTCGGCGCGGCCGATATAG -3'
hcmvUL141/279rev/His/EcoRI (long-long)
5'- CCGGAATTCTCAGTGATGGTGATGGTGATGTCCCCGAGTGGCCCAGGG -3'
huTR2-Fc/58for/EcoRI
5'- CCGGAATTCCAACAAGACCTAGCTCCCCA -3'
huTR2-Fc/184rev/PstI
5'- CCGCTGCAGGCCTGATTCTTTGTGGACACA -3'
hcmvUL141-Fc/37for/EcoRI
5'- CCGGAATTCGACATTGCCGAAAAGATGTGG -3'
hcmvUL141-Fc/247rev/PstI (middle)
5'- CCGCTGCAGGCAGTCGCCGGGGAGCC -3'
hcmvUL141-Fc/273rev/PstI (long)
5'- CCGCTGCAGAGACATTCCGGTGTCTATGTC -3'
