# Supplementary material for: Structure of Human Cytomegalovirus UL141 Binding to TRAIL-R2 Reveals Novel, Non-canonical Death Receptor Interactions
Source: PLoS Pathog. 2013 Mar 21;9(3):e1003224. doi: 10.1371/journal.ppat.1003224 (PMC3605307; doi:10.1371/journal.ppat.1003224)
Supplement: Table S3 — List of multi-site mutation primers. (PDF) [file ppat.1003224.s010.pdf]

Table S3. List of multi-site mutation primers.

Single-stranded multi-site (3-4) mutation primers for Quick Change II Multi-site Kit
**R133A_N134A_TRAIL-R2–Fc**
5'-GTCCCTGCACCACGACCGCAGCCACAGTGTGTCAGTGCG-3'
**Y103_TRAIL-R2–Fc**
5'-TCCTGCAAATATGGACAGGACGCTAGCACTCAGTGGAATGAC-3'
**L110A_F112A_L114A_R115A_TRAIL-R2–Fc**
5'-CTCACTGGAATGACGCCCTTGCCTGCGCGGCCTGCACCAGGTGTG-3'
**D109_ TRAIL-R2–Fc**
5'-CAGGACTATAGCACTCACTGGAATGCCCTCCTTTTCTGCTTG-3'
**E147A_D148A_P150A_E151A_TRAIL-R2–Fc**
5'-GCACCTTCCGGGAAGCAGCTTCTGCTGCGATGTGCCGGAAGTG-3'
**M152A_R154A_K155A_TRAIL-R2–Fc**
5'-AGAAGATTCTCCTGAGGCGTGCGCGGCGTGCCGCACAGGGTGT-3'
**V167A_TRAIL-R2–Fc**
5'-TGTCCCAGAGGGATGGTCAAGGCCGGTGATTGTACACCC-3'
**W173A_I176A_V179A_TRAIL-R2–Fc**
5'-GATTGTACACCCGCGAGTGACGCCGAATGTGCCCACAAAGAATCA-3'
**E78_G79_TRAIL-R2–Fc**
5'-AGGTCCAGCCCCTCAGCGGCATTGTGTCCACCTGGACACCAT-3'
